# The predictive skill of convolutional neural networks models for disease forecasting

**DOI:** 10.1371/journal.pone.0254319

**Published:** 2021-07-09

**Authors:** Kookjin Lee, Jaideep Ray, Cosmin Safta

**Affiliations:** 1 Computing, Informatics and Decision Systems Engineering, Arizona State University, Tempe, AZ, United States of America; 2 Extreme-Scale Data Science and Analytics, Sandia National Laboratories, Livermore, CA, United States of America; 3 Quantitative Modeling and Analysis, Sandia National Laboratories, Livermore, CA, United States of America; Fuzhou University, CHINA

## Abstract

In this paper we investigate the utility of one-dimensional convolutional neural network (CNN) models in epidemiological forecasting. Deep learning models, in particular variants of recurrent neural networks (RNNs) have been studied for ILI (Influenza-Like Illness) forecasting, and have achieved a higher forecasting skill compared to conventional models such as ARIMA. In this study, we adapt two neural networks that employ one-dimensional temporal convolutional layers as a primary building block—temporal convolutional networks and simple neural attentive meta-learners—for epidemiological forecasting. We then test them with influenza data from the US collected over 2010-2019. We find that epidemiological forecasting with CNNs is feasible, and their forecasting skill is comparable to, and at times, superior to, plain RNNs. Thus CNNs and RNNs bring the power of nonlinear transformations to purely data-driven epidemiological models, a capability that heretofore has been limited to more elaborate mechanistic/compartmental disease models.

## Introduction

Seasonal influenza resulted in 9–45 million illnesses, between 140k–180k hospitalizations, and between 12k–61k deaths in the United States annually since 2010. It is the subject of intensive and continuous surveillance by the US Center for Disease Control (CDC) [1]. Forecasting influenza outbreaks is important as it allows us to plan for medical resource demands [2]. To address this problem, CDC has been focusing its efforts on *Flu Forecasting* [3], of which the goal is to predict the disease dynamics, e.g. predicting the timing, peak, and intensity of flu season, so that its impact can be reduced. Since the competition, “Predict the Influenza Season Challenge” (see [4]), hosted by CDC in 2013, CDC has encouraged researchers to develop models to accurately predict influenza activities for a number of weeks ahead. Along with traditional mechanistic modeling approaches, there have been many studies on developing statistical forecast models using historical flu activity data: auto-regressive modeling [5], Google Flu Trend [6], Google search data [7, 8]), and refined models using structural spatio-temporal synchronicities [9, 10], to name a few.

More recently, with advancements in deep learning and the availability of high-spatial-resolution data, i.e., ten US HHS-regions-level data (regions defined by the United States Department of Health & Human Services) and state-level data for all US states, there have been many studies on applying deep learning techniques for flu forecasting [11–17]. Among several artificial neural network architectures dedicated to handling time series, there are largely two types of architectures that have been extensively studied for time-series predictions: recurrent neural networks (RNNs), which consist of shared and recurring units, and convolutional neural networks (CNNs), which consist of layers of convolutional operations. RNNs are defined as a family of neural networks for processing “sequential data” [18] and have shown a significant impact in applications such as language modeling [19, 20], machine translation [21], and polyphonic music modeling [22]. RNNs and their variants also have been actively explored in flu prediction studies [11–17].

CNNs are often considered to be specialized neural networks for processing images, exploiting local correlations in space. CNNs, however, also have been applied to sequence modeling in many applications including speech recognition [23] and natural language processing [24–28]. More recently, variants of CNNs that are specially designed for sequence modeling using operations, “temporal” convolutions, have demonstrated an improved performance in audio synthesis [29], machine translations [25, 30], and other sequence modeling [31] applications compared to earlier neural network (NN) models. In particular, the extensive experiments in Ref. [31] demonstrated that relative simple temporal convolution architectures could exhibit substantially longer memory than RNNs. Despite these successes, temporal convolutions have rarely been explored in modeling infectious disease models and consequently, little is known about their effectiveness in forecasting disease evolution, vis-à-vis RNNs.

In this study, we model flu forecasting as a sequence modeling task, explore different NN architectures, namely variants of RNNs and CNNs, and then compare the performance of all models with extensive experiments. Following [6, 7, 10], we utilize CDC data, gathered over nine years, on the percentage of the number of influenza-like illness (ILI) patients over the total number of outpatient visits, which is denoted by %ILI; this information can be used as a proxy of the flu activity in the population and helps hospital officials allocate appropriate resources in preparation for increased patient visits to hospital facilities. This study considers only the state-level %ILI from CDC without any external data (e.g., external factors that might affect the flu activity or additional internet data that can be utilized for forecasting). We decided against using COVID-19 data in this study for two reasons: (1) There are only three waves’ worth of data, which does not provide a sufficient sampling of the diversity of outbreak variation and (2) the waves are significantly affected by external forcings e.g., the different types of population mixing during the 2020 spring, summer and winter waves in the US, public health measures that changed with each wave etc., which would have to be modeled separately, as they are exogenous to the data. In contrast, the ILI data reflects the same seasonality and public health measures for each year, making them intrinsic properties of the data set and thus “learnable” by data-driven models.

Both statistical time-series models [32] e.g., ARIMA, and mechanistic/compartmental models [33] are widely used in forecasting epidemics. Compartmental models require one to model population mixing and disease phenomenology (e.g., incubation, prodrome, existence and role of asymptomatic individuals etc.) which can be difficult in case of novel diseases. However, modern information technology allows the (very) quick and comprehensive collection of basic epidemiological data e.g., detected or diagnosed cases, at the country-, regional- and often, city-scale, as evidenced during the ongoing COVID-19 pandemic [34, 35]; purely data-driven methods could be used for forecasting and practical resource planning scenarios even without fully understanding the disease dynamics. This raises the question whether more complex data-driven models, such as CNNs and RNNs, can be trained on big data to provide better forecasts than linear ARIMA models. RNNs, in fact, have been used for forecasting COVID-19 [36], and comparisons of RNN-based forecasting for influenza have shown that neural networks perform far better than ARIMA models [15, 17], or other baseline machine learning methods such as support vector machine (SVM) and AdaBoost [11]. In this paper, thus, we focus only on deep-learning-based prediction models and we investigate whether CNNs, one-dimensional temporal convolutions, in particular, could be used for the same purpose.

The paper is structured as follows. In Section “Methods and materials” we review modern recurrent neural networks and two other neural networks based on temporal convolutions and describe datasets in detail. In Section “Results and discussion” we describe training strategies for neural networks and we present results of experiments of one-week ahead predictions and multi-week ahead predictions. Finally, in Section “Conclusion”, we present our conclusions.

## Methods and materials

### Methods—Sequence modeling via deep learning

Assume that we have a sequence of input data {*x*_1_, …, *x*_*t*_} with the time index *t*, which can be obtained, for example, from measurements, and we wish to predict future sequence, {*x*_*t*+1_, *x*_*t*+2_, …} via sequence modeling. In general form, sequence modeling tries to learn a function *f*(⋅; Θ) such that

$$\begin{array}{c} \left\{o_{1},\ldots ,o_{n_{\text{out}}}\right\}=f\left(x_{1},\ldots ,x_{n_{\text{in}}};\Theta \right), \end{array}$$

where Θ is a set of parameters to be learned, and $\left\{o_{1},\ldots ,o_{n_{\text{out}}}\right\}$ is a sequence of outputs. Here, *n*_in_ and *n*_out_ are not necessarily the same. In a supervised learning setting, there are target (or reference) variables $\left\{y_{1},\ldots ,y_{n_{\text{out}}}\right\}$, on which the model *f*(⋅;Θ) can be trained to produce outputs $\left\{o_{1},\ldots ,o_{n_{\text{out}}}\right\}$ to match target variables $\left\{y_{1},\ldots ,y_{n_{\text{out}}}\right\}$. In a forecasting scenario, where we attempt to predict future data, the target variables can be set as, for example, *y*_1_ = *x*_*t*+1_, *y*_2_ = *x*_*t*+2_ for 2-weeks ahead prediction. Given input data and target variables, an optimal set of parameters can be learned by minimizing a loss function $L(y_{1},\ldots ,y_{n_{\text{out}}},o_{1},\ldots ,o_{n_{\text{out}}})$, which computes the discrepancy between the output $\left\{o_{1},\ldots ,o_{n_{\text{out}}}\right\}$ and the target $\left\{y_{1},\ldots ,y_{n_{\text{out}}}\right\}$ in a certain measure.

The parameterized function *f*(⋅;Θ) can take various forms such as statistical models or deep-learning-based models. In this study, we will model *f*(⋅;Θ) using deep-learning-based models, such as recurrent neural networks (RNNs), sequence-to-sequence (or encoder-decoder type) RNN variants, and neural networks based on temporal-causal convolutions. Our goal is to perform a comprehensive empirical evaluation of the performance of various types of neural networks for near-term forecasting of influenza-like illness. Below, we briefly review each of the deep-learning-based models considered in this study.

### Standard recurrent neural networks (RNN)

**Fig 1:**
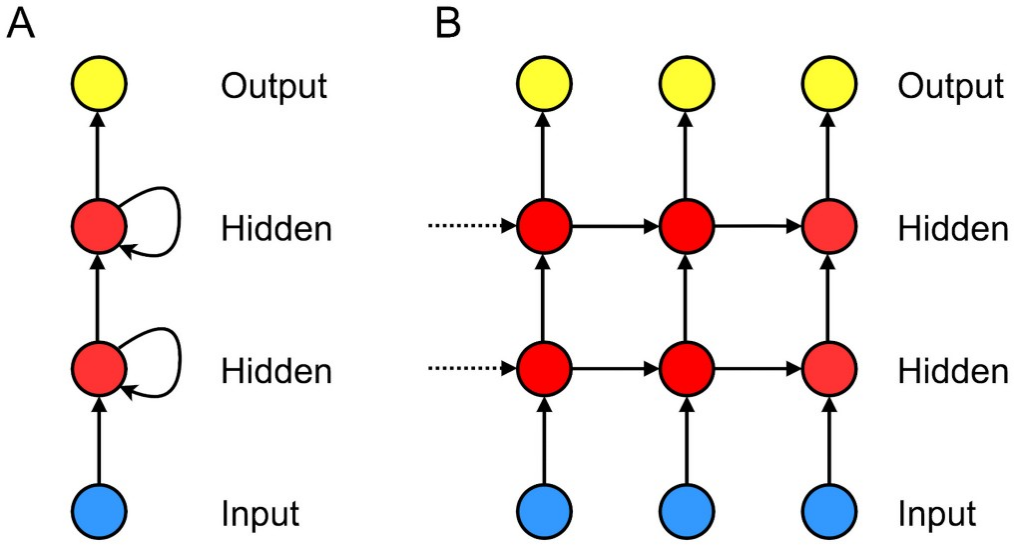
RNN architecture. An example RNN architecture with two hidden layers: A. a folded representation and B. an unfolded representation. The blue circles correspond to an input layer, the red circles correspond to hidden layers, and the yellow circles correspond to an output layer.

A recurrent neural network (RNN) [37] is a type of neural network designed to process a sequence of values and, thus, is well-suited for predicting time-sequences. At time index *τ*, RNNs typically operate on a sequence of data *x*_*τ*_, update hidden states $h_{\tau }^{\left(1\right)},h_{\tau }^{\left(2\right)},\ldots$, and produce an output *o*_*τ*_ by applying the same function parameterized by a set of neural network weights:

$$\begin{array}{c} o_{\tau },h_{\tau }^{\left(1\right)},h_{\tau }^{\left(2\right)},\ldots =\text{RNN}\left(x_{\tau },h_{\tau -1}^{\left(1\right)},h_{\tau -1}^{\left(2\right)},\ldots ;\Theta \right), \end{array}$$ (1)

where ***h***^(*ℓ*)^ denotes the *ℓ*-th hidden layer. Fig 1 depicts an example RNN architecture with two hidden layers and Eq (1) describes a folded representation of RNN, shown in the left frame of Fig 1.

Hidden layers and outputs of RNNs are computed in sequence. Assume a RNN architecture with one hidden layer. Then, starting from a given initial hidden state $h_{0}^{\left(1\right)}$, the forward pass of the RNN computes subsequent hidden layers by applying an affine transformation to a current input and the previous hidden states followed by a nonlinear activation, e.g., $h_{\tau }^{\left(1\right)}=\sigma \left(W_{xh}x_{\tau }+W_{hh}h_{\tau -1}^{\left(1\right)}+b\right)$, where *W*_*xh*_, *W*_*hh*_, and ***b*** are the learnable parameters and *σ* is a nonlinear activation function.

### Modern RNNs

Standard RNNs typically suffer from vanishing/exploding gradient problems [38–40]. To cope with this difficulty, architectures using gating mechanisms including long short-term memory (LSTM) [39] and gated recurrent unit (GRU) [41] have been proposed and extensively used in several applications. The core idea is to create paths whose gradients do not vanish or explode as RNN cells become far apart.

#### Long short-term memory (LSTM)

In LSTM recurrent networks, the above issue is resolved by adding LSTM *cells*, which have an internal recurrence to update cell *states*, generating paths where the gradient can flow over longer durations [39], as opposed to standard RNNs that only have recurrence over the hidden states. In addition, by introducing gating mechanisms which typically consists of *input*, *output*, and *forget* gates, the flow of information can be effectively controlled [39, 42]. As the name indicates, the *input* gate controls the amount of information gets transferred from input to the current LSTM cell, the *output* gate controls the amount of information gets transferred to output, and the *forget* gate controls the extent to which internal cell state remains unchanged. When the sigmoid function is used, each gate produces a value between [0, 1], where 0 and 1 are two extreme values indicating that no or all information will be passed, respectively. S1 Appendix contains a more detailed description of LSTMs.

#### Gated recurrent unit (GRU)

GRU is another gating mechanism without internal cell states and with a small number of gates; GRU consists of *reset* and *update* gates [41]. The *reset* gate controls information flow choosing which parts of the previous state to pass to generate a new target state. Then the *update* gate controls the extent to which the previous state remains and the new target state replaces the previous state to generate a new state.

Compared to LSTM, GRU has a simpler architecture: it does not have a cell state and it operates only on two gates (as opposed to three gates in LSTM). In general, LSTM is known to be strictly more expressive and it has been demonstrated that LSTM outperforms GRU on many different applications [43, 44]. In certain tasks, however, GRU performs similar to LSTM [45], or performs even better in small datasets [22]. We refer readers to S1 Appendix for details on LSTM, GRU, and their comparisons.

### Sequence-to-sequence (Seq2Seq) model

**Fig 2:**
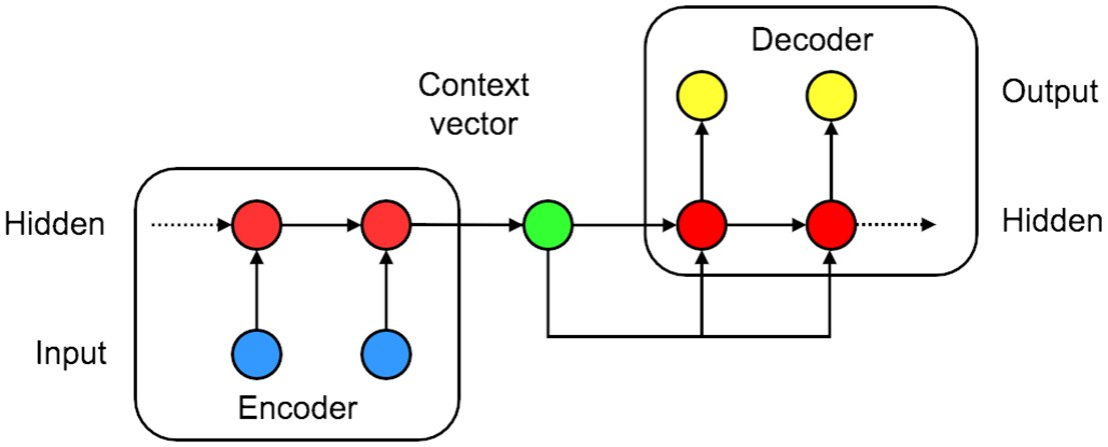
Seq2Seq architecture. An example Seq2Seq architecture with one hidden layer in the encoder and the decoder. The blue circles correspond to an input layer, the red circles correspond to hidden layers, the yellow circles correspond to an output layer, and the light green circle in the middle correspond to a context vector.

Next, we consider a sequence-to-sequence (Seq2Seq) model (or, often called encoder-decoder model): the Seq2Seq architecture is designed to train a mapping from an input sequence to an output sequence, where the input and the output sequences do not necessarily have the same length [41, 46]. For handling variable-length sequences, the Seq2Seq architecture consists of an encoder and a decoder, which are typically RNN-type architectures (with LSTM cells or GRUs). Because the input and the output of ILI forecasting can be two variable-length sequences, the Seq2Seq architecture is a naturally good fit. The encoder processes an input sequence to produce a single vector called a *context vector*, which is typically a function of the last hidden layer of the encoder. Then the decoder generates an output sequence conditioned on the single context vector. Fig 2 depicts an illustration of a Seq2Seq architecture.

### Convolutional networks

**Fig 3:**
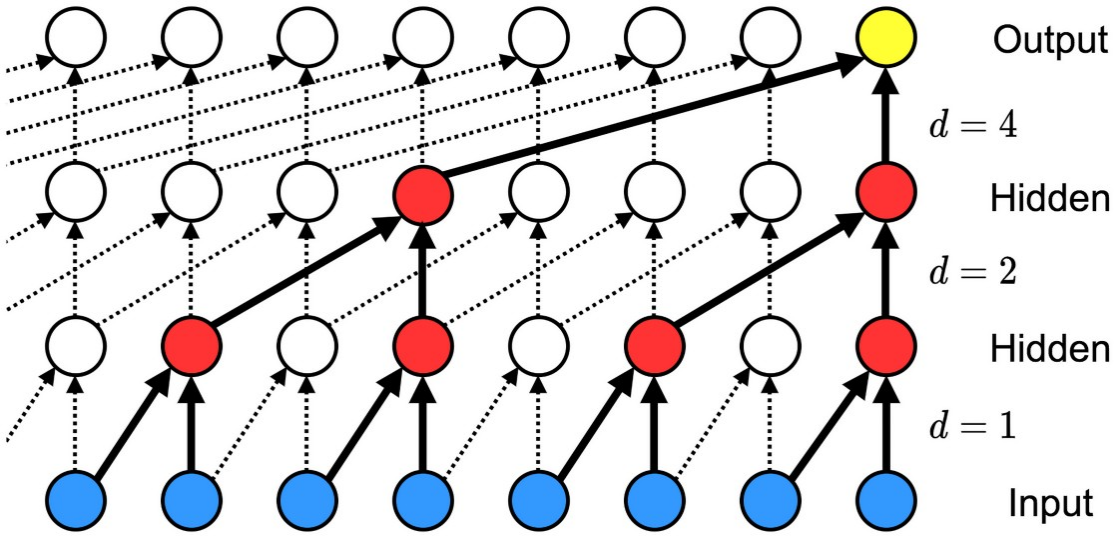
TCN architecture. A temporal convolutional network with layers corresponding to exponentially increasing dilation factors *d* = 1, 2, 4. The blue circles correspond to an input layer, the red circles correspond to hidden layers, and the yellow circles correspond to an output layer.

As an alternative set of sequence modeling approaches, we present two neural networks: temporal convolutional networks and simple neural attentive meta-learners, of which the main component is a temporal convolution (TC) layer. The TC layer refers to a one-dimensional causal convolution layer; an input to this layer is the 1D time-sequenced data, which may consists of multi channels, same as in an input to a regular 1D convolutional layers, and as the term, “causal”, indicates, an output of the TC layer at time *t*, *o*_*t*_, is produced by applying convolution kernel filters to the input data up to time *t*, {…, *x*_*t*−1_, *x*_*t*_}. To utilize a longer history of the input data without making the depth of neural network too deep, a sequence of dilated convolutions with increasing dilation factors can be used as in Ref. [29, 47]. In a typical setting, the dilation factor starts with *d* = 1 and increases exponentially, i.e., *d* = *O*(2^*ℓ*^) at level *ℓ* of the network. Thus, the receptive field of the network can be controlled by the kernel size *k* and the dilation factor *d*. As for RNNs, TC layers can handle arbitrary-length input sequence. Fig 3 illustrates an example of TC layers with several dilation factors.

#### Temporal convolutional networks (TCNs)

Among several neural network models based on the temporal convolutions, we explore the temporal convolution networks (TCNs) [31] in this study since TCNs provide a relatively simple, but flexible architecture. Moreover, TCNs employ residual blocks [48], which we denote by ResBlock, for stabilization of forward/backward passes of deep neural network architectures. Each ResBlock consists of (1) two layers of dilated causal convolution, where each layer is followed by weight normalization, ReLU [49], and dropout, and (2) the identity mapping from the input to the block (optionally, a 1 × 1 convolutional layer can be employed to match the input and the output shapes so that the element-wise summation can be performed). We refer readers to S1 Appendix for details of TCNs.

#### Simple neural attentive meta-learner (SNAIL)

As described above, TCNs handle long sequences by employing exponentially increasing dilation factors. This may lead to coarser access to inputs and, consequently, bounded network capacity. To resolve this issue, a simple neural attentive meta-learner (SNAIL) [50] combines temporal (causal) convolutions with a soft attention mechanism that is similar to Ref. [51]. SNAIL interleaves TC layers with attention layers so that the model can learn to (1) extract features from the current and previous input features/data via the TC layers and (2) identify more important input features/data over other elements in long sequences via the attention layers.

**Fig 4:**
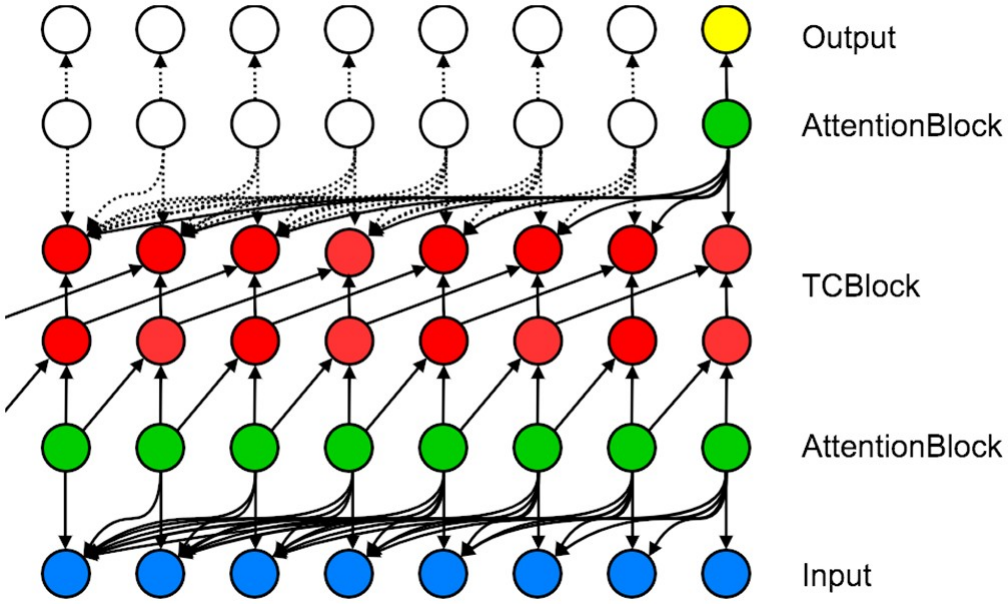
SNAIL architecture. A schematic view of SNAIL with one TCBlock and two AttentionBlocks. The blue circles correspond to an input layer, the red circles correspond to hidden layers, the yellow circles correspond to an output layer, and the green circles correspond to attention layers.

These two main ingredients of SNAIL can be summarized by two separate functions, TCBlock and AttentionBlock. In TCBlock, a series of TC layers with exponentially increasing dilation factors is used; at each TC layer, an output is computed using the gated activation function [29, 52] (as opposed to the simple TCNs) and then is concatenated with an input. In AttentionBlock, a causal attention mechanism is employed to point out at which points in the input sequence should be more emphasized. The term “causal” indicates that the attention mechanism only looks at the current time input data and its previous time input data. Fig 4 depicts a schematic view of SNAIL with one TCBlock and two AttentionBlocks. We refer readers to S1 Appendix for details of TCBlock and AttentionBlock.

### Materials

#### Datasets

The Influenza Division at the Center for Disease Control (CDC) reports a weekly U.S. influenza surveillance data (FluView); in particular, we are interested in information on outpatient visits to health care providers for influenza-like illness (ILI), which is collected through the U.S. Outpatient Influenza-like Illness Surveillance Network (ILINet). ILINet consists of outpatient healthcare provider in all 50 states and records the total number of patient visits and the number of those patients with ILI symptoms, which are defined as combinations of fever (temperature over 100°F), a cough, and/or a sore throat without a known cause other than influenza.

Our particular interest lies in the percentage of the number of ILI patients over the total number of outpatient visits, which is denoted by %ILI (% unweighted ILI as opposed to % weighted ILI, where the percentage is weighted on the basis of state population). This information is available per state for each week and can be downloaded from the application, FluView Interactive [53].

#### Preprocessing

We downloaded state-level %ILI from week 40 of 2010 to week 39 of 2019 (9 seasons), and preprocessed it to keep %ILI only in the influenza season (i.e., week 40 of a given year to week 20 of a subsequent year). Consequently, each influenza season consists of 33 weeks, except for 2014–2015 season, which has 34 weeks due to the 53rd week in 2014. Among the 50 states, Florida is excluded from the study because there is no data reported. We rescale the data to have values in range [0, 1] using min-max scaling. Note that predictions made by each neural network are then scaled back to the original range by applying the inverse of the min-max scaling operator in a post-processing step and the resuling quantities are used to compute performance metrics, which will be introduced in the next Section. Moreover, we generate binary mask, which has the same length as the % ILI data, to indicate missing, unreported, or zero-value recorded weekly report.

**Fig 5:**
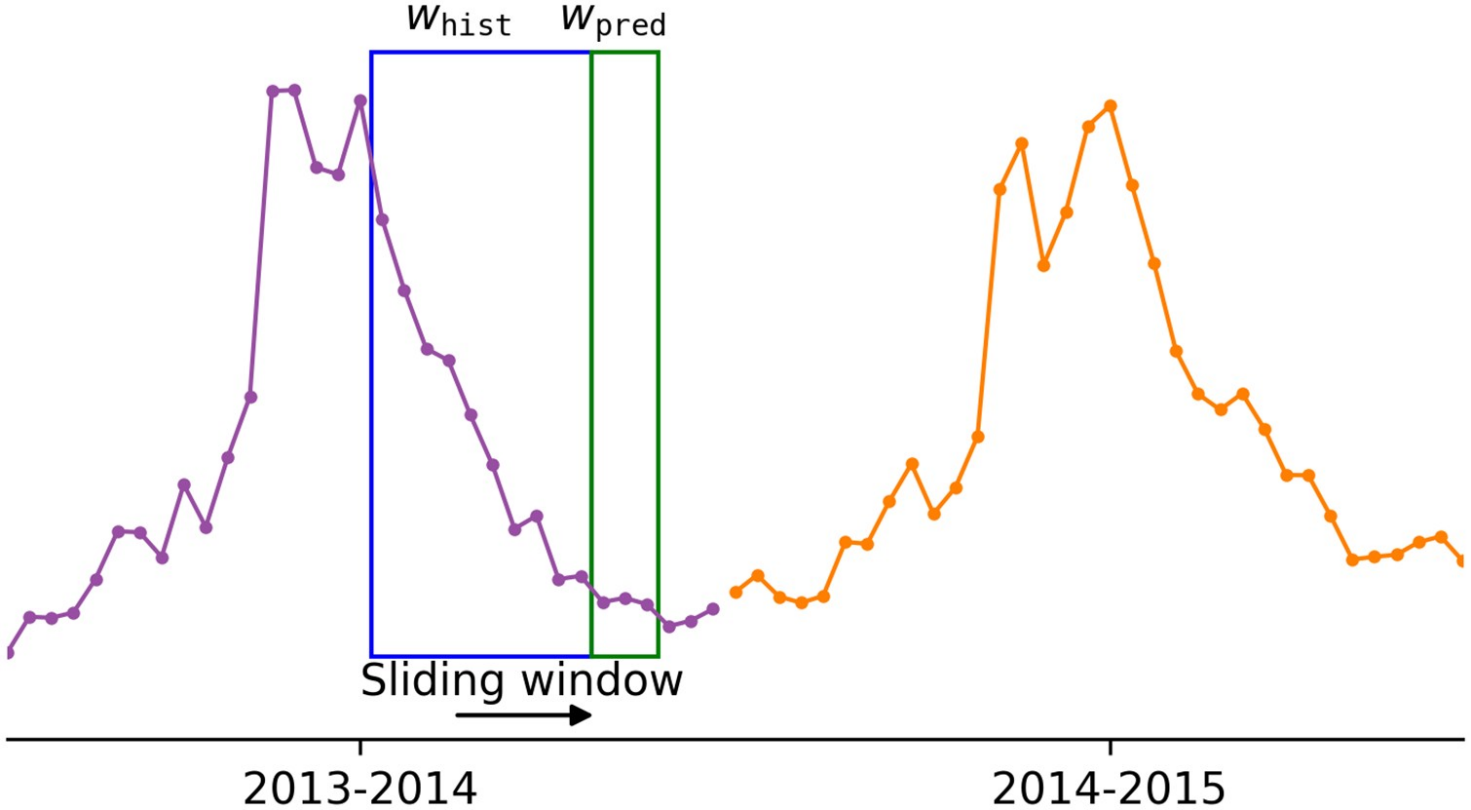
CA %ILI curve. An example illustration of applying a sliding window to a %ILI curve of California from the 2013–2014 season to the 2014–2015 season.

Following the preprocessing step shown in Ref. [17], we concatenate 9 seasons of %ILI per each state and use a fixed-length sliding window to generate subsequences for supervised-learning settings (see Fig 5). The window consists of two subwindows of lengths, *w*_hist_ and *w*_pred_ (in weeks), and sequence models are expected to make predictions on *w*_pred_ weeks of %ILI given *w*_hist_ weeks of historical observations on %ILI.

We then split the data into training/validation/test sets. The training set, the validation set, and the test set consist of subsequences obtained from 2010 to 2017 (7 seasons), from the 2017–2018 season, from the 2018–2019 season, respectively. For *w*_pred_-weeks-ahead predictions, the validation set and the test set are designed to include subsequences of which the last data point belongs to the 2017–2018 season and the 2018–2019 season, respectively.

## Results and discussion

We now assess the performance for %ILI forecasting for the neural networks described in the previous section. We construct all neural network architectures using PyTorch 1.12 [54]. Before presenting the experimental results, we describe neural network training strategies employed in this work and performance metrics used to evaluate the trained neural networks on the test dataset.

### Training neural networks

For training neural networks, we consider the mean squared error (MSE) as the loss function, and minimize the loss function by employing a gradient-based optimization method [55]. In particular, we employ a variant of stochastic gradient descent method called Adamax [56] with the initial learning rate 10^−2^ and we set the mini-batch size to 50. The maximum number of epochs is set of 50. At each epoch, training is performed by computing gradients of the loss on the training set and the validation loss is computed on the validation set. After the maximum number of epochs, the best performing network weights on the validation set are chosen to try the model on the test data. Moreover, we use an early-stopping strategy; the training stops if there is a certain number of epochs where the optimizer fails to find improvements in validation loss. From our empirical findings, in general, the early-stopping strategy does not help to generalize the performance of the RNN variants within the pre-specified maximum number of epochs, whereas the strategy helps to generalize the performance of the TC variants. Thus, we use the early-stopping strategy only for TCNs and SNAILs, and set the number of consecutive epochs to 3 for early-stopping criterion.

We also employ a grid search for optimal hyperparameter values. Although there are more advanced hyperparameter search algorithms such as Bayesian hyperparameter optimization [57], we use grid search to put more emphasis on the impact of these hyperparameters on the performances of each models and attempt to draw interpretations on impact of these changes on model improvements.

### Performance metrics

For assessing performances of each neural networks, we consider four different metrics to measure the discrepancy between the predicted sequence and the target sequence: root mean-square error (RMSE), mean absolute percentage error (MAPE), relative error measured in the Euclidean norm (L2E), and Pearson Correlation (PCORR). These metrics are define as:

$$\begin{array}{cc} \text{RMSE} & ={\left(\frac{1}{N}\sum_{i=1}^{N}{\left(x_{i}-{\tilde{x}}_{i}\right)}^{2}\right)}^{1/2},\\ \text{MAPE} & =\frac{1}{N}\sum_{i=1}^{N}\frac{|x_{i}-{\tilde{x}}_{i}|}{x_{i}+1}\times 100,\\ \text{L2E} & =\frac{{\left(\sum_{i=1}^{N}{\left(x_{i}-{\tilde{x}}_{i}\right)}^{2}\right)}^{1/2}}{{\left(\sum_{i=1}^{N}x_{i}^{2}\right)}^{1/2}},\\ \text{PCORR} & =\frac{\sum_{i=1}^{N}\left(x_{i}-m_{x}\right)\left({\tilde{x}}_{i}-m_{\tilde{x}}\right)}{\sqrt{\sum_{i=1}^{N}{\left(x_{i}-m_{x}\right)}^{2}\sum_{i=1}^{N}{\left({\tilde{x}}_{i}-m_{\tilde{x}}\right)}^{2})}}, \end{array}$$

where *x*_*i*_ and ${\tilde{x}}_{i}$, *i* = 1, …, *N* denote the ground truth %ILI and the approximate quantity produced by neural networks, respectively, and *m*_*x*_ refers to the mean of *x*_*i*_, *i* = 1, …, *N*.

RMSE and L2E compute the averaged and the relative sum of squared errors, respectively. MAPE computes the errors in L1-norm and then reports the error as a percentage. Following [15], the denominator is smoothed by adding 1 to avoid zero values. Lastly, PCORR measures how correlated two sequences are and the resulting quantity lies between [-1, 1]. The closer PCORR is to 1, the more positively correlated the two sequences are. Note that L2E and MAPE are relative measures, whereas RMSE is not: RMSE measures the squared differences of *x*_*i*_’s where *x*_*i*_ < 16 in test sets.

### One-week-ahead predictions results

For this experiment, we use varying lengths of historical observations *w*_hist_ = {16, 32, 64, 128} to make a one-week-ahead predictions, i.e., *w*_pred_ = 1. We will explore the performance of models discussed above with the metric introduced in the previous section. Unless otherwise specified, we compute one of each error for 49 states separately and then average each error over 49 states. We begin by comparing RNN-LSTM and RNN-GRU.

#### Long short-term memory and gated recurrent unit

For both RNN-LSTM and RNN-GRU, we consider the same supervised learning setting. As described in the preprocessing section, we consider the subsequences of length *w*_hist_+*w*_pred_. We denote the *i*-th input data sequence by $x_{1}^{\left(i\right)},\ldots ,x_{w_{\text{hist}}}^{\left(i\right)}$, where $x_{\tau }^{\left(i\right)}$ is used as the input to the *τ*-th step of the RNNs. At the *τ*-th RNN step, the network produces output $o_{\tau }^{\left(i\right)}$, which attempts to match $y_{\tau }^{\left(i\right)}=x_{\tau +1}^{\left(i\right)}$ so that, in the last RNN step, the quantity that the network tries to predict is produced. We train both RNN-LSTM and RNN-GRU by minimizing the MSE.

**Fig 6:**
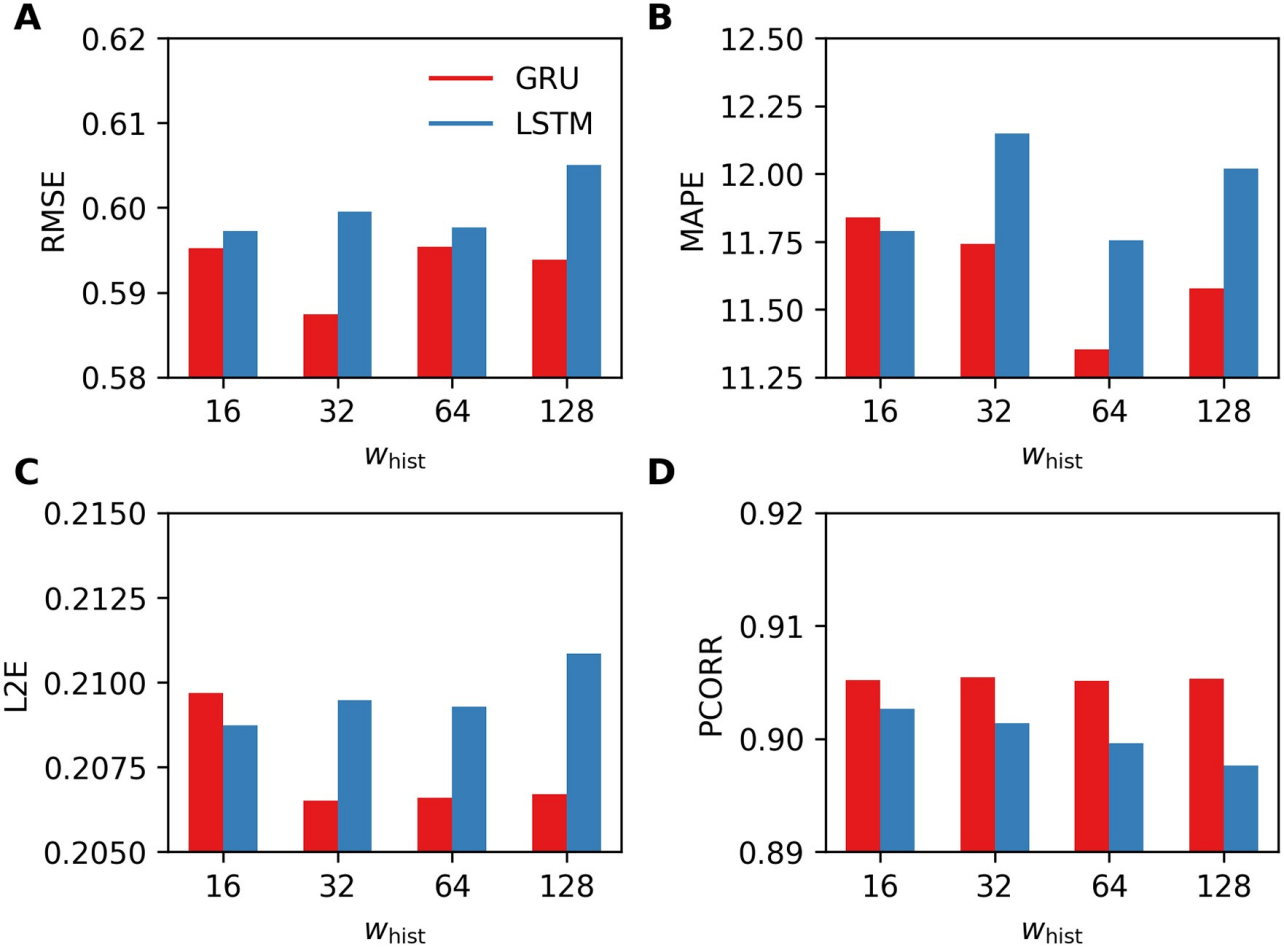
Four performance metrics for one-week-ahead predictions obtained by using RNN-LSTM and RNN-GRU. A. RMSE, B. MAPE, C. L2E, and D. PCORR. Smaller values are preferable for RMSE, MAPE, L2E, and larger values are preferable for PCORR.

In the first experiment, we consider RNN-LSTM and RNN-GRU with two hidden layers. For both RNNs, the first layer consists of 32-dimensional hidden units (and 32-dimensional cell state in LSTM) and the second layer consists of 16-dimensional hidden units (and 16-dimensional cell state in LSTM). For implementation, we use a series of LSTMCell and GRUCell, respectively. Then the output of the last layer is connected to a dense network with one hidden layer, which consists of 100 units, followed by a ReLU nonlinear activation. We have tested these two RNNs on the datasets obtained by varying the lengths of historical observations *w*_hist_ = {16, 32, 64, 128}. Fig 6 reports the four performance metrics computed using the test set and, in most cases, RNN-GRU results in better performances.

**Fig 7:**
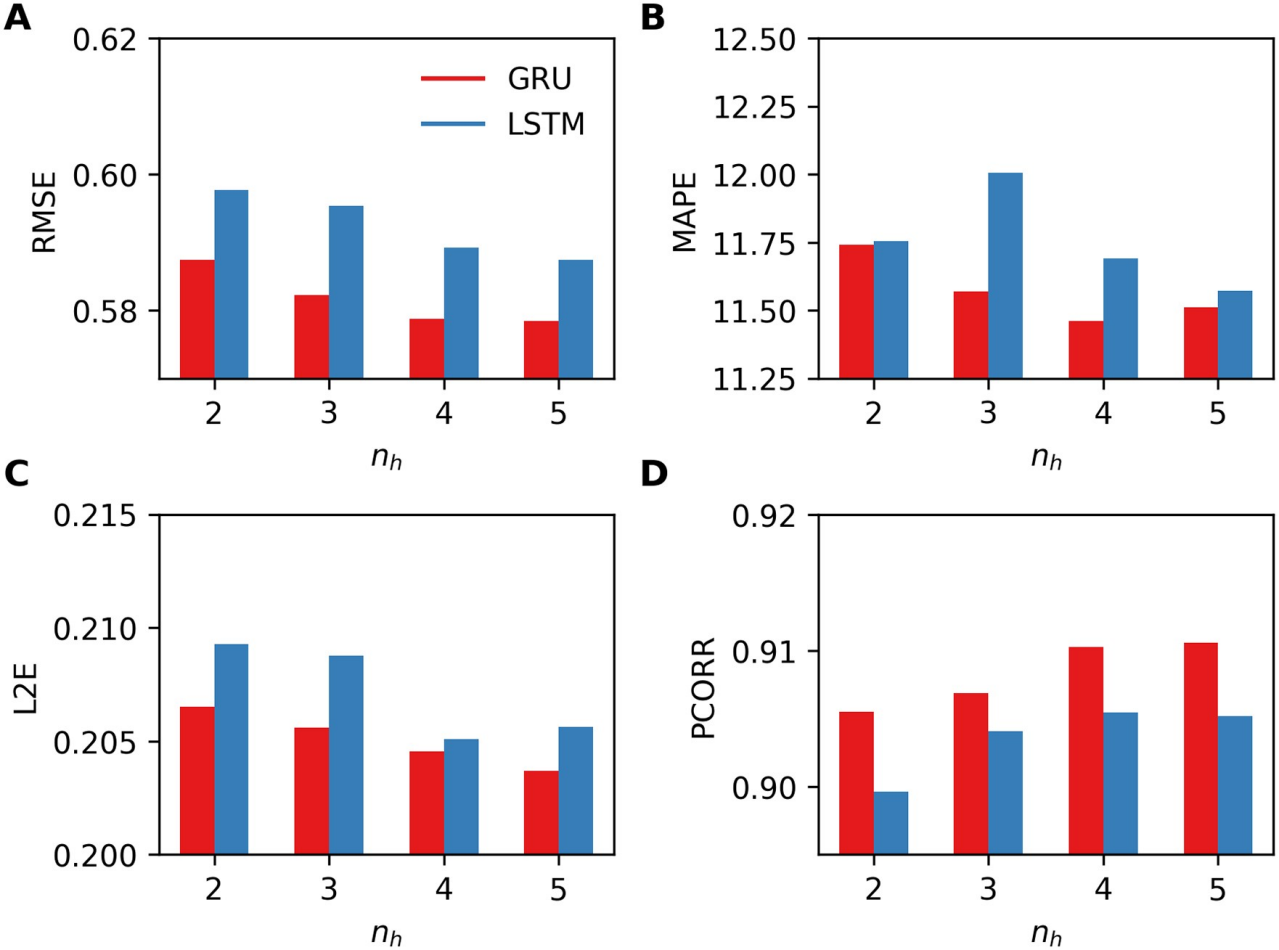
Four performance metrics for one-week-ahead predictions obtained by using RNN-LSTM and RNN-GRU for varying number of layers *n*_*h*_ = {2, 3, 4, 5}. A. RMSE, B. MAPE, C. L2E, and D. PCORR. Smaller values are preferable for RMSE, MAPE, L2E, and larger values are preferable for PCORR.

Next we consider RNNs with varying number of LSTM layers and GRU layers as *n*_***h***_ = {2, 3, 4, 5}. We fixed the size of hidden units in the last layer to be 16 and those of other layers to be 32. We tested the new networks for varying lengths of historical observations *w*_hist_ = {16, 32, 64, 128} as described above. From these experiments, we observe that RNN-LSTM performs best with *w*_hist_ = 64 and RNN-GRU performs best with *w*_hist_ = 32. Fig 7 reports the performance metrics measured for the LSTM and the GRU in their best settings. For both RNNs, we can observe that all four errors decrease, except for MAPE of LSTM, as *n*_***h***_ increases. Although other results are not reported, we also have observed that increasing *w*_hist_ to 128 significantly decreases the performance of both LSTM and GRU. Overall, RNN-GRU outperforms RNN-LSTM in all error metrics and, thus, we consider only GRU in the following experiments.

#### Sequence-to-sequence models

Seq2Seq-type networks consist of an encoder and a decoder and both of them are modeled as RNN-GRU in this study. As for the previous settings used in the comparisons of RNN-LSTM and RNN-GRU, the *i*-th input sequence is denoted by $x_{1}^{\left(i\right)},\ldots ,x_{w_{\text{hist}}}^{\left(i\right)}$ and the encoder RNN network operates on this input sequence to produce a context vector. Then the decoder receives the last element in the input sequence (i.e., $x_{w_{\text{hist}}}^{\left(i\right)}$) as its input as well as the context vector to produce output $o_{1}^{\left(i\right)}$, which attempts to match $y_{1}^{\left(i\right)}=x_{w_{\text{hist}}+1}^{\left(i\right)}$.

The encoder and the decoder are designed to have a symmetric architecture in terms of the number of hidden layers. For instance, if the encoder consists of two hidden layers of unit sizes 32 and 16, the decoder consists of two hidden layers of unit sizes 16 and 32. As in the previous experiments with LSTM and GRU, we have tested the Seq2Seq architecture with varying number of GRU layers by setting *n*_***h***_ = {2, 3, 4, 5}: the last hidden layer of the encoder and the first hidden layer of the decoder are of dimension 16, and the other hidden layers are of dimension 32. We again consider varying lengths of historical observations *w*_hist_ = {16, 32, 64, 128}.

**Fig 8:**
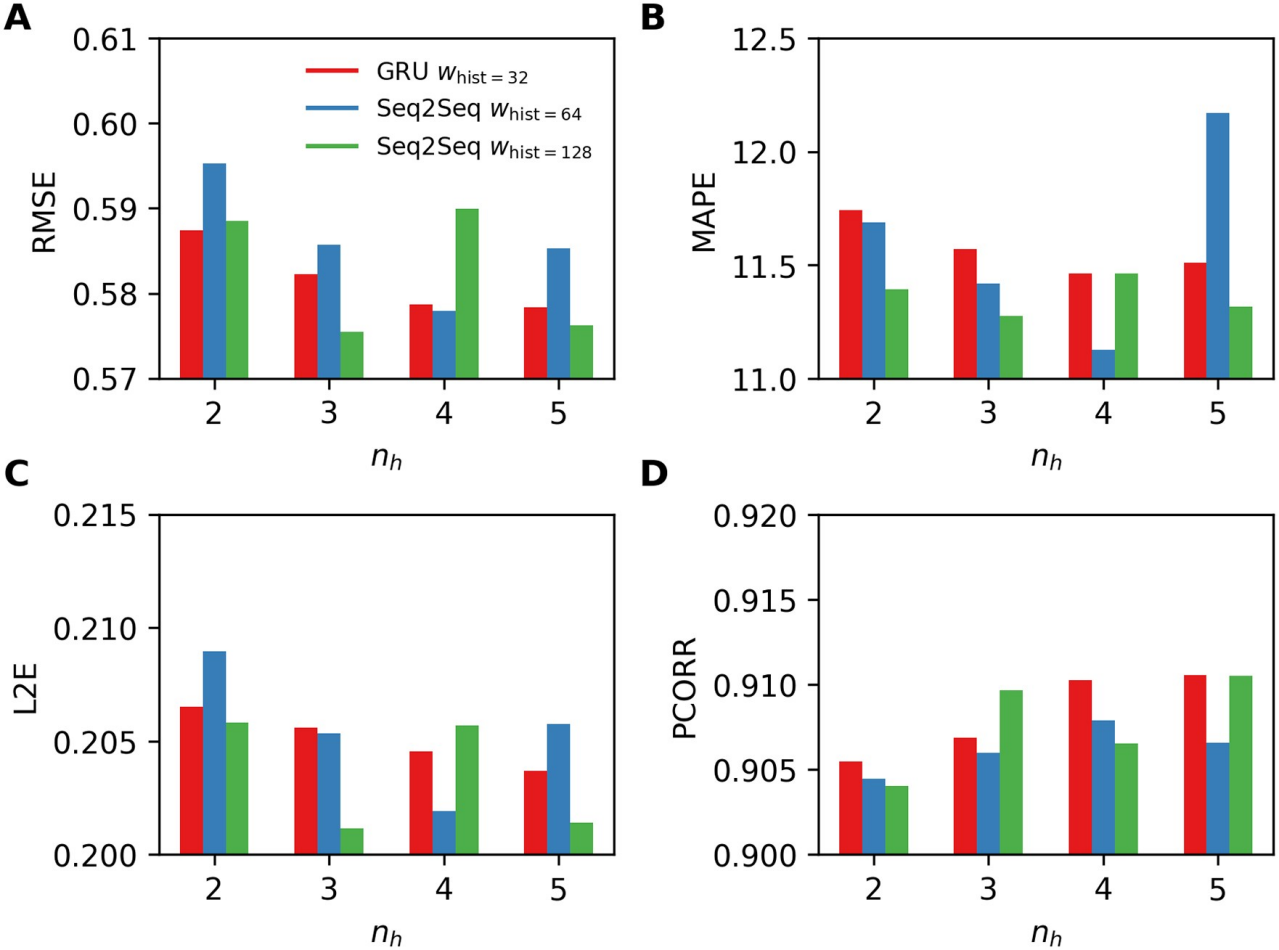
Four performance metrics for one-week-ahead predictions obtained by using RNN-GRU and the Seq2Seq model for varying number of layers *n*_*h*_ = {2, 3, 4, 5}. A. RMSE, B. MAPE, C. L2E, and D. PCORR. Smaller values are preferable for RMSE, MAPE, L2E, and larger values are preferable for PCORR. Only the best performing values of *w*_hist_ are presented.

In Fig 8, we present results obtained by using the Seq2Seq architecture with two best performing values of *w*_hist_ = {64, 128}, and compare those results with the best results obtained by RNN-GRU (*w*_hist_ = 32) presented in Fig 7. The experiments show that the Seq2Seq architecture performs better with longer sequences (i.e., *w*_hist_ = {64, 128}) and, as shown in the experiments with RNN-GRU, performs better with the larger networks (i.e., *n*_***h***_ ≥ 3), in general; we note that there is a single exception *n*_***h***_ = 4 and *w*_hist_ = 128. By comparing the results of Seq2Seq with GRU in Fig 7, we observe that the Seq2Seq architectures outperform the standard RNN-GRU in many cases, and the Seq2Seq architecture with *n*_***h***_ = 3 and *w*_hist_ = 64 produces the best results.

As an alternative to the RNN-GRU decoder, we also have explored different choices of the network architecture for decoder including neural ordinary differential equations (NODEs [58]) and augmented NODEs (ANODEs) [59]. NODEs have demonstrated their ability to learn latent dynamics of complex physical processes [58, 60] and have been successfully used for time-sequence modeling for various applications [61–64]. Motivated by these successes, we design another Seq2Seq model, of which the encoder is RNN-GRU and the decoder is NODE or ANODE; the NDOE or the ANODE takes the context vector as an initial condition and computes hidden states at pre-specified time steps *τ* = {1, …, *w*_hist_, *w*_hist_ + 1, …, *w*_hist_ + *w*_pred_}. We again refer readers to S1 Appendix for the technical details of NODE and ANODE. Here, our intention is to train NODEs/ANODEs to learn the underlying dynamics of the evolution of %ILI and make prediction from the learned dynamics.

Seq2Seq with ANODE decoder, in general, performs better than Seq2Seq with NODE decoder. However, both Seq2Seq models with NODE and ANODE decoders perform worse than Seq2Seq with GRU in all four error metrics. Table 1 shows the four performance metrics for one-week-ahead predictions computed by using the Seq2Seq models with RNN-GRU decoder, NODE decoder and ANODE decoder: the best performing Seq2Seq model with ANODE decoder achieves about 3–6% worse performance in RMSE, MAPE, and L2E and about 1% worse performance in PCORR. The best performing network architecture for ANODE (NODE, resp) consists of a 6-layer (5-layer, resp) RNN-GRU encoder with hidden dimension sizes 32 (for internal layers) and 16 (for the outermost layer), and an ANODE decoder with hidden dimension 16, augmented by an extra 16 dimensional vector (no augmentation, resp) and the dynamics of the hidden dimension is described by a 6-layer (5-layer, resp) multi-layer perceptron (MLP) with width 32.

**Table 1:** Four performance metrics for one-week-ahead predictions obtained by using the Seq2Seq models with RNN-GRU decoder, NODE decoder, and ANODE decoder. The results of the best performing hyperparameters are shown.

decoder type	RNN-GRU	NODE	ANODE
RMSE	0.5756	0.6065	0.5939
MAPE	11.14%	12.10%	11.74%
L2E	0.2012	0.2146	0.2100
PCORR	0.9104	0.9028	0.9043

We believe that both NODE and ANODE perform worse than RNN-GRU decoder because only the context vector (i.e., an initial state of ODEs) is given to the decoder to produce an output sequence including future predictions. This is opposed to RNNs, where the *τ*-th data can be fed into the network as an input to the *τ*-th RNN step and can be used to produce the (*τ*+1)-th prediction. Thus, to make accurate predictions with NODE and ANODE, underlying dynamics of %ILI describing the long-term dependency has to be learned very accurately, which is typically very difficult to achieve (due to e.g., noise in measurements, limited number of observations). Along with this reason, since RNN-LSTM or GRU is the dominant choice in many previous studies [11–16], we will consider only the Seq2Seq model with RNN-GRU as our baseline for comparison in the following experiments.

#### Temporal convolutional networks

Next we discuss results obtained by TCN. In this experiment, we consider the same supervised learning settings for the input and the target data, i.e., the *i*-th input data sequence is ${\left\{x_{\tau }^{\left(i\right)}\right\}}_{\tau =1}^{w_{\text{hist}}}$ and the *i*-th target data sequence is ${\left\{y_{\tau }^{\left(i\right)}\right\}}_{\tau =1}^{w_{\text{hist}}}$, where *y*_*τ*_ = *x*_*τ*+1_ and consider the same loss function, the MSE.

For TCN, there are three hyperparameters that we can tune for TC layers: the number of ResBlock *n*_R_, the number of kernel filters *n*_*k*_, and the size of kernels *k*. In the following experiments, we vary these hyperparameters as *n*_R_ = {3, 4, 5, 6, 7, 8}, *n*_*k*_ = {4}, and *k* = {2, 4, 8}. Note that we employ the same number of kernel filters for all ResBlocks (e.g., if *n*_*k*_ = 4, all TC layers in all ResBlocks have 4 kernel filters) because we observe that changing this parameter has a negligible impact to the performances in the near-term %ILI prediction. Lastly, as in the previous experiments, we vary lengths of historical observations *w*_hist_ = {16, 32, 64, 128}.

For the choices of the number of residual blocks *n*_R_ = {3, 4}, there seem be no clear trend or differences between the performances of TCNs for all considered hyperparameters, *k* = {2, 4, 8} and *w*_hist_ = {16, 32, 64, 128}. For *n*_R_ = {5, 6, 7, 8}, the TCNs perform better with longer subsequences *w*_hist_ = {64, 128}. We believe this is caused by the fact that increasing the number of ResBlocks results in larger dilation factors *d* and, consequently, larger receptive fields, which does not have significant impacts on shorter subsequences (i.e., *w*_hist_ = {16, 32}), but have significant impacts on longer subsequences (i.e., *w*_hist_ = {64, 128}).

**Fig 9:**
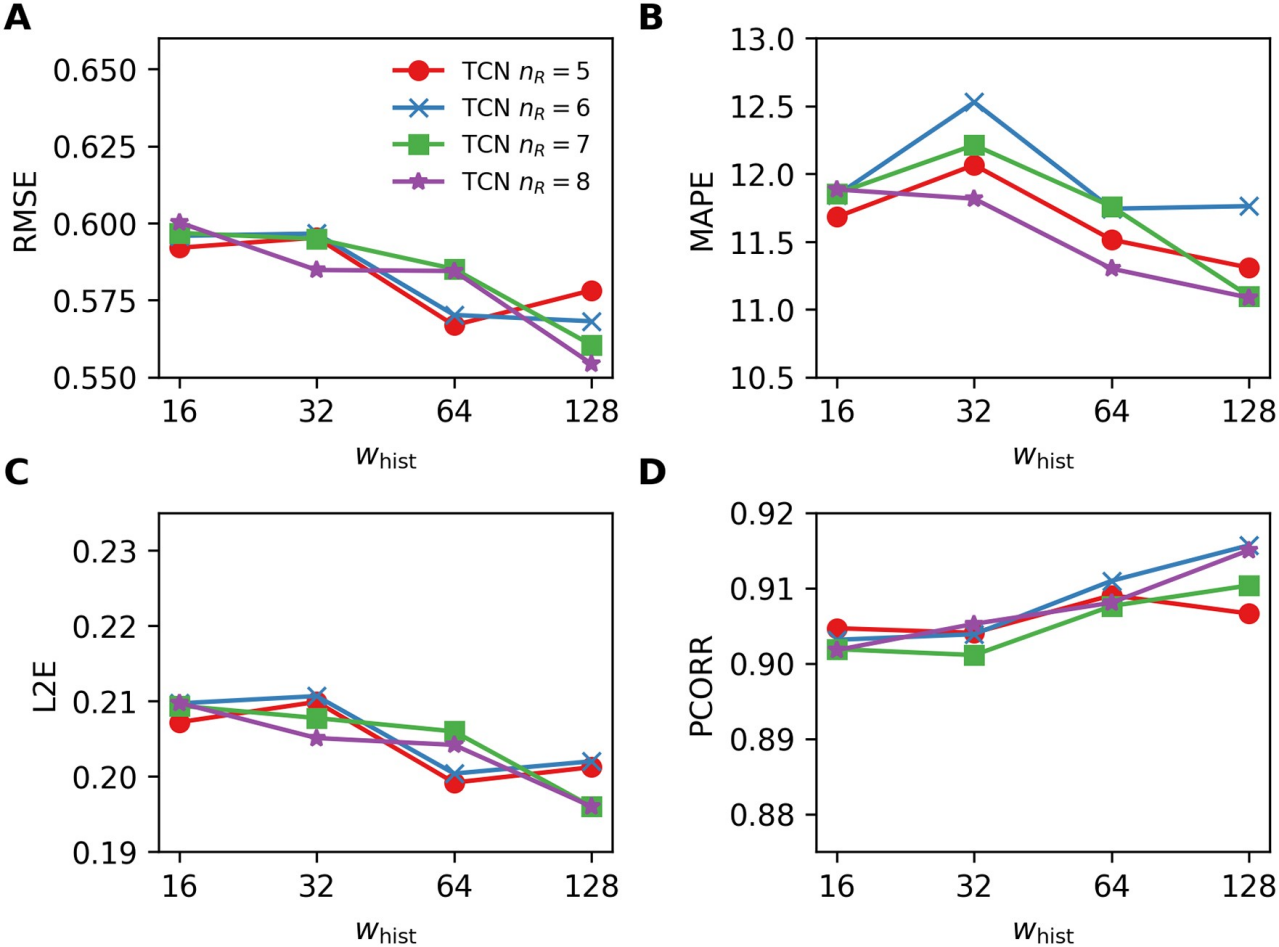
Four performance metrics for one-week-ahead predictions obtained by using the TCNs for varying number of residual blocks *n*_R_ = {5, 6, 7, 8} with the kernel size *k* = 4. A. RMSE, B. MAPE, C. L2E, and D. PCORR. Smaller values are preferable for RMSE, MAPE, L2E, and larger values are preferable for PCORR.

Fig 9 shows the results obtained by using the TCNs for varying number of residual blocks *n*_R_ = {5, 6, 7, 8} with the kernel size *k* = 4. The figures essentially show that RMSE, MAPE, and L2E tend to decrease and PCORR tend to increase as *w*_hist_ becomes longer. From the experiments of the TCNs with the kernel size *k* = {2, 8} and *n*_R_ = {5, 6, 7, 8}, we observe the same trend (i.e., improved performances with longer *w*_hist_) in most cases.

#### Simple neural attentive meta-learner

Next, we present experimental results obtained by using SNAIL. Again, we consider the same supervised learning setting for the input and the target data, i.e., the *i*-th input data sequence is ${\left\{x_{\tau }^{\left(i\right)}\right\}}_{\tau =1}^{w_{\text{hist}}}$ and the *i*-th target data sequence is ${\left\{y_{\tau }^{\left(i\right)}\right\}}_{\tau =1}^{w_{\text{hist}}}$, where *y*_*τ*_ = *x*_*τ*+1_ and we minimize the MSE.

We follow the same network architecture considered in the original paper [50]; there are three AttentionBlocks interleaved with two TCBlocks followed by a 1 × 1 convolutional layers. For each TCBlock, there are three hyperparameters for TC layers, the number of TC layers *n*_TC_, the number of kernel filters *n*_*k*_, and the size of kernels *k*; we consider the same choices of the hyperparameters for the two TCBlocks. From the empirical experience that we obtained from the previous experiments with TCNs, we choose the values of the hyperparameters as follows: *n*_TC_ = {6, 7, 8}, *n*_*k*_ = {4, 8}, and *k* = {2, 4, 8}. For each AttentionBlock, there are two hyperparameters, the size of the attention keys *n*_key_ and the size of the attention values *n*_value_. We vary the value of the first parameter as *n*_key_ = {16, 32} and fix the value of the second parameter as *n*_value_ = 32.

**Fig 10:**
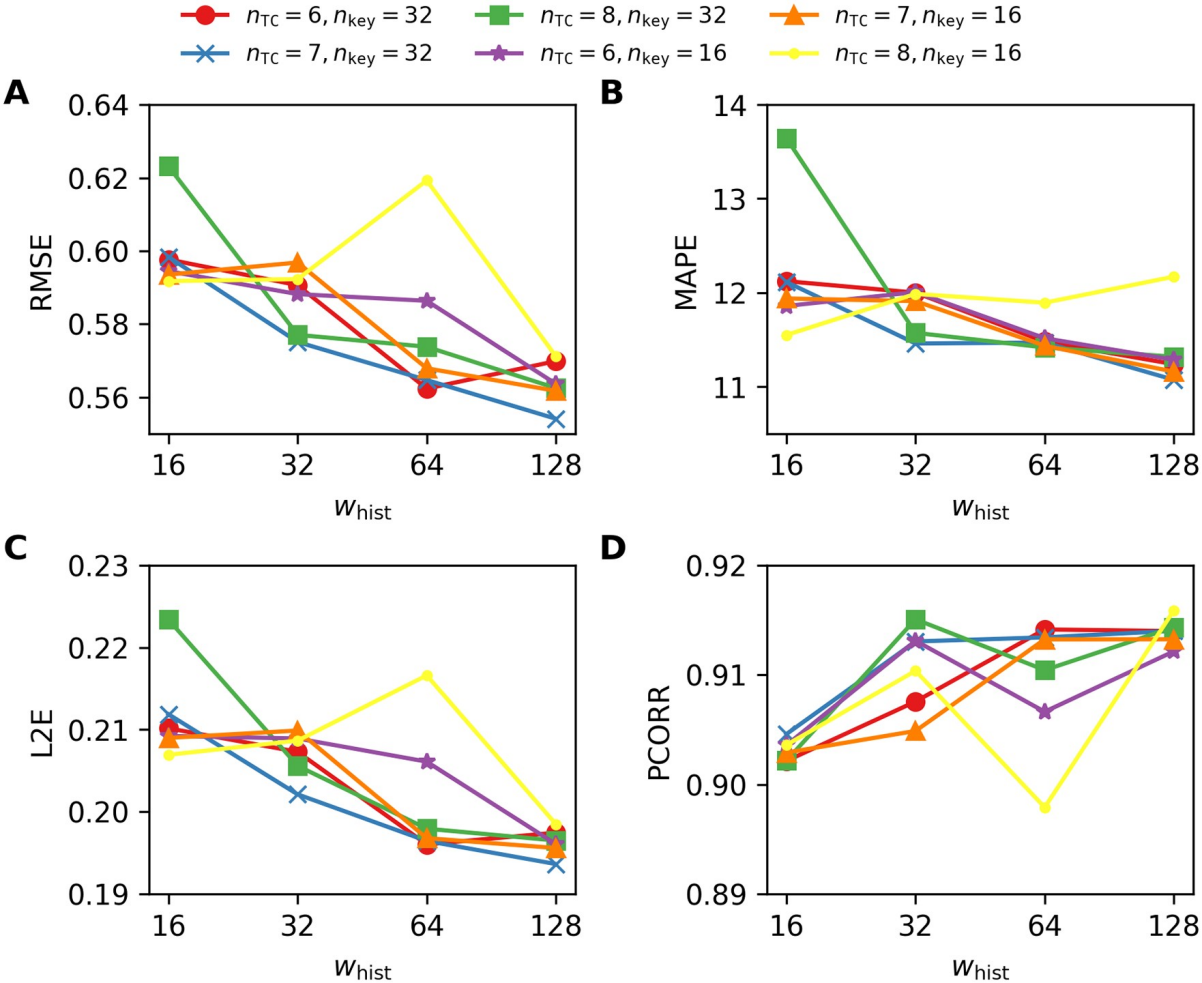
Four performance metrics for one-week-ahead predictions obtained by using the SNAILs for varying number of TC layers *n*_TC_ = {6, 7, 8} with the kernel size *n*_key_ = {16, 32}. A. RMSE, B. MAPE, C. L2E, and D. PCORR. Smaller values are preferable for RMSE, MAPE, L2E, and larger values are preferable for PCORR.

As shown in the experimental results of TCNs, the SNAIL architectures tend to perform better with longer sub-sequences (i.e., *w*_hist_ = {64, 128}). Fig 10 depicts the results obtained by using the SNAIL architectures with the kernel size *k* = 4 for varying *n*_TC_ and *n*_key_, and shows that the best performances are achieved with *w*_hist_ = 128 in most cases (with the exception depicted in the yellow curve). We also observe that the SNAIL architectures with *k* = {2, 8} and *n*_*k*_ = 8, of which experimental results are not shown, perform the best with *w*_hist_ = 128 in most cases.

#### Performance comparisons between Seq2Seq, TCN, and SNAIL

We now compare the three sequence models discussed above: Seq2Seq architecture with GRU cells, TCN, and SNAIL. For each performance metric, we pick the best performing neural network configurations (i.e., network architectures and their hyperparameters). Table 2 lists the neural network configurations of each considered neural networks that perform the best for each performance metric. Note that for RMSE and L2E, the same neural network configurations are used to achieve the best performances.

**Table 2:** Neural network configurations: The number of GRU layers *n*_*h*_, the number of ResBlocks *n*_R_, the number of TCBlocks *n*_TC_, the kernel size *k*, the number of kernel filters *n*_*k*_, and the size of key *n*_key_ in SNAIL. For TCN and SNAIL, the value of *w*_hist_ is omitted as setting *w*_hist_ = 128 yields the best results. For TCN, *n*_*k*_ = 4.

	Seq2Seq	TCN	SNAIL
RMSE	(*n*_***h***_, *w*_hist_) = (3, 128)	(*n*_R_, *k*) = (8, 4)	(*n*_TC_, *k*, *n*_*k*_, *n*_key_) = (7, 4, 4, 32)
MAPE	(*n*_***h***_, *w*_hist_) = (4, 64)	(*n*_R_, *k*) = (8, 2)	(*n*_TC_, *k*, *n*_*k*_, *n*_key_) = (6, 2, 8, 32)
L2E	(*n*_***h***_, *w*_hist_) = (3, 128)	(*n*_R_, *k*) = (8, 4)	(*n*_TC_, *k*, *n*_*k*_, *n*_key_) = (7, 4, 4, 32)
PCORR	(*n*_***h***_, *w*_hist_) = (5, 128)	(*n*_R_, *k*) = (7, 4)	(*n*_TC_, *k*, *n*_*k*_, *n*_key_) = (7, 4, 4, 32)

**Fig 11:**
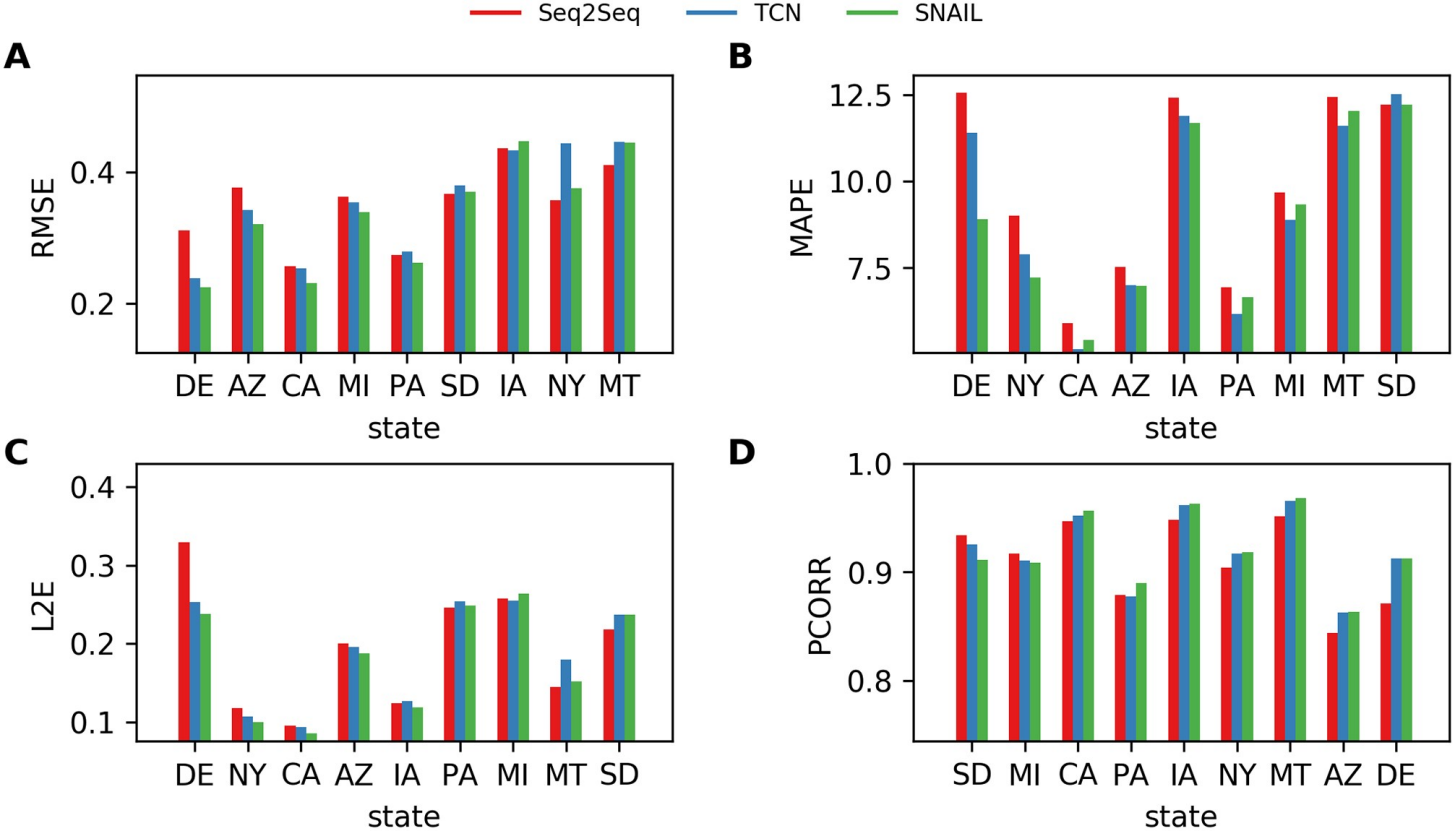
The best performances achieved by Seq2Seq, TCN, and SNAIL for one-week-ahead forecasts. A. RMSE, B. MAPE, C. L2E, and D. PCORR. Four performance metrics, RMSE, MAPE, L2E, and PCORR, are measured for each state. In the horizontal axis, the states are placed in a decreasing order w.r.t. the relative performance of SNAIL against Seq2Seq. Results of the nine selected states, {AZ, CA, DE, IA, MA, MT, NY, PA, SD} are depicted.

Fig 11 reports the four performance metrics measured in each state using Seq2Seq, TCN, and SNAIL; for better presentation, only nine states are selected for visualization, (Arizona, AZ; California, CA; Delaware, DE;, Iowa, IA; Massachusetts, MA; Montana, MT; New York, NY; Pennsylvania, PA; South Dakota, SD; henceforth, {AZ, CA, DE, IA, MA, MT, NY, PA, SD}). The states were chosen as they span the full range of performance metrics. The states in Fig 11A–11C are presented in a decreasing order w.r.t. the relative performance of SNAIL against Seq2Seq (i.e., $\frac{\text{Performance}\;\text{of}\;\text{SNAIL}}{\text{Performance}\;\text{of}\;\text{Seq2Seq}}$). Thus, the relative performance of SNAIL against Seq2Seq decreases going from left to right. For a more comprehensive comparison between all the states of the US, see Ref. [65].

Table 3 shows the four performance metrics computed using the best performing neural network configurations (Table 2). Along with the results of neural networks, we also present the results of more traditional approaches: persistence model, autoregressive (AR) model and autoregressive integrated moving average (ARIMA) model. For AR, we have tested three different lag values 16, 32, and 64. We report only the results of the best performing setting (i.e., lag is 16, AR(16)). For ARIMA, following Refs. [14, 17], we consider the choice of parameter values (*p*, *d*, *q*) = (3, 0, 3), where *p* and *q* refer to the orders of the autoregressive model and the moving-average model, and *d* refers to the degree of differencing. For the earlier works on epidemics modeling with ARIMA, we refer readers to Refs. [66, 67]. Each performance metric is measured for each state and then averaged over 49 states. For all four performance metrics, both TCN and SNAIL outperform Seq2Seq; SNAIL performs slightly better than TCN. Recall that MAPE and L2E are relative measures and MAPE is a percentage. RMSE is an absolute measure, where the input data in the test set lies in [0, 16].

**Table 3:** The best performances achieved by Persistence, AR(16), ARIMA(3,0,3), Seq2Seq, TCN, and SNAIL for one-week-ahead forecasts. The performance metrics are averaged over 49 states. The symbols ↓ and ↑ indicate that the lower and higher values correspond to the better performance.

	Persistence	AR(16)	ARIMA(3,0,3)	Seq2Seq	TCN	SNAIL
RMSE (↓)	0.6218	0.6030	0.5871	0.5756	0.5543	**0.5541**
MAPE (↓)	12.37	12.86	11.90	11.14	10.98	**10.86**
L2E (↓)	0.2150	0.2231	0.2147	0.2012	0.1955	**0.1937**
PCORR (↑)	0.8950	0.9035	0.9017	0.9104	0.9157	**0.9161**

**Fig 12:**
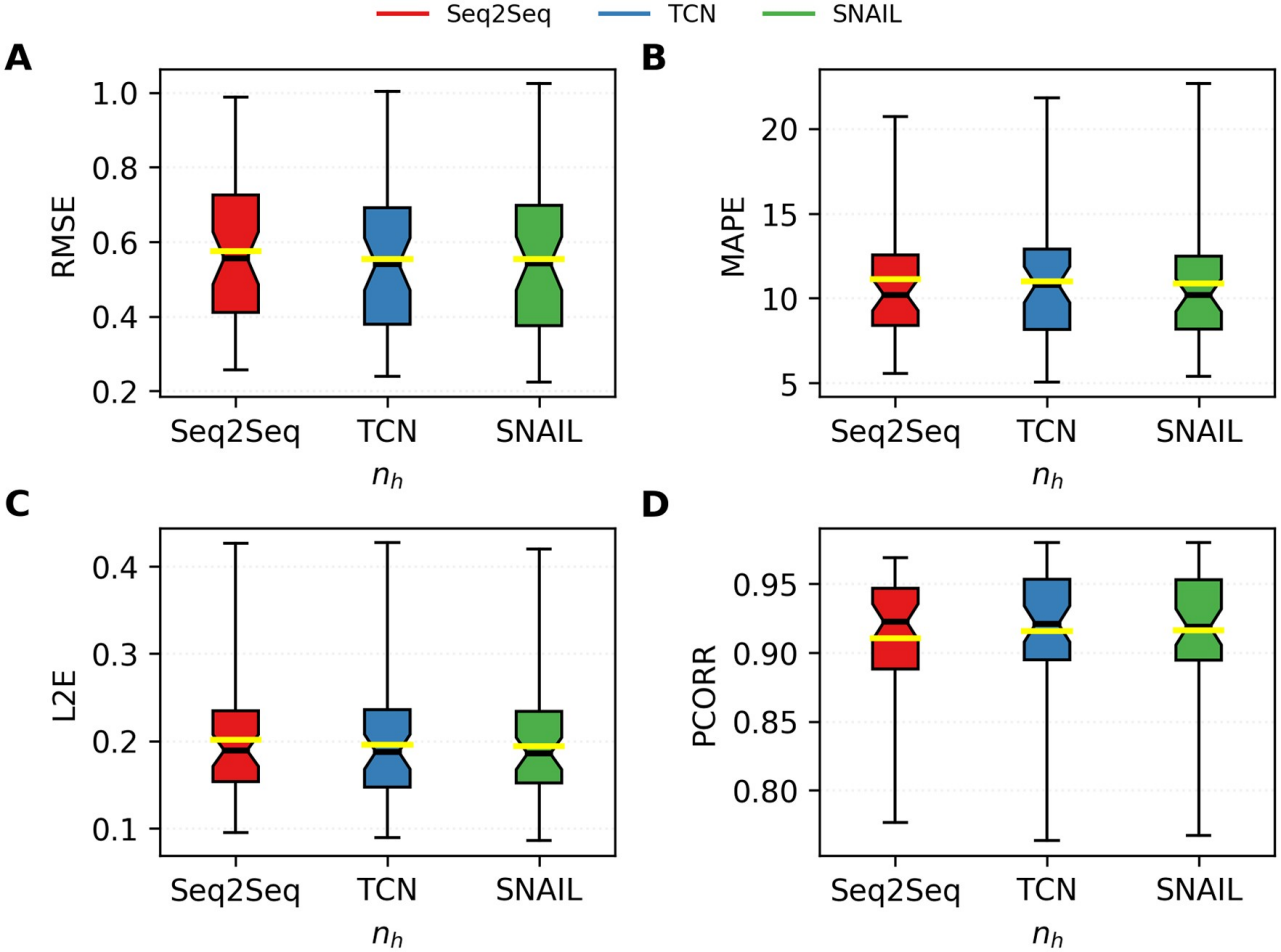
The boxplots of all four performance metrics measured by using Seq2Seq, TCN, and SNAIL. A. RMSE, B. MAPE, C. L2E, and D. PCORR. The boxplots summarize statistics of the predictions made for every 49 states. The middle black and yellow lines indicate medians and means, respectively, and the boxes indicates the interquartile ranges.

**Fig 13:**
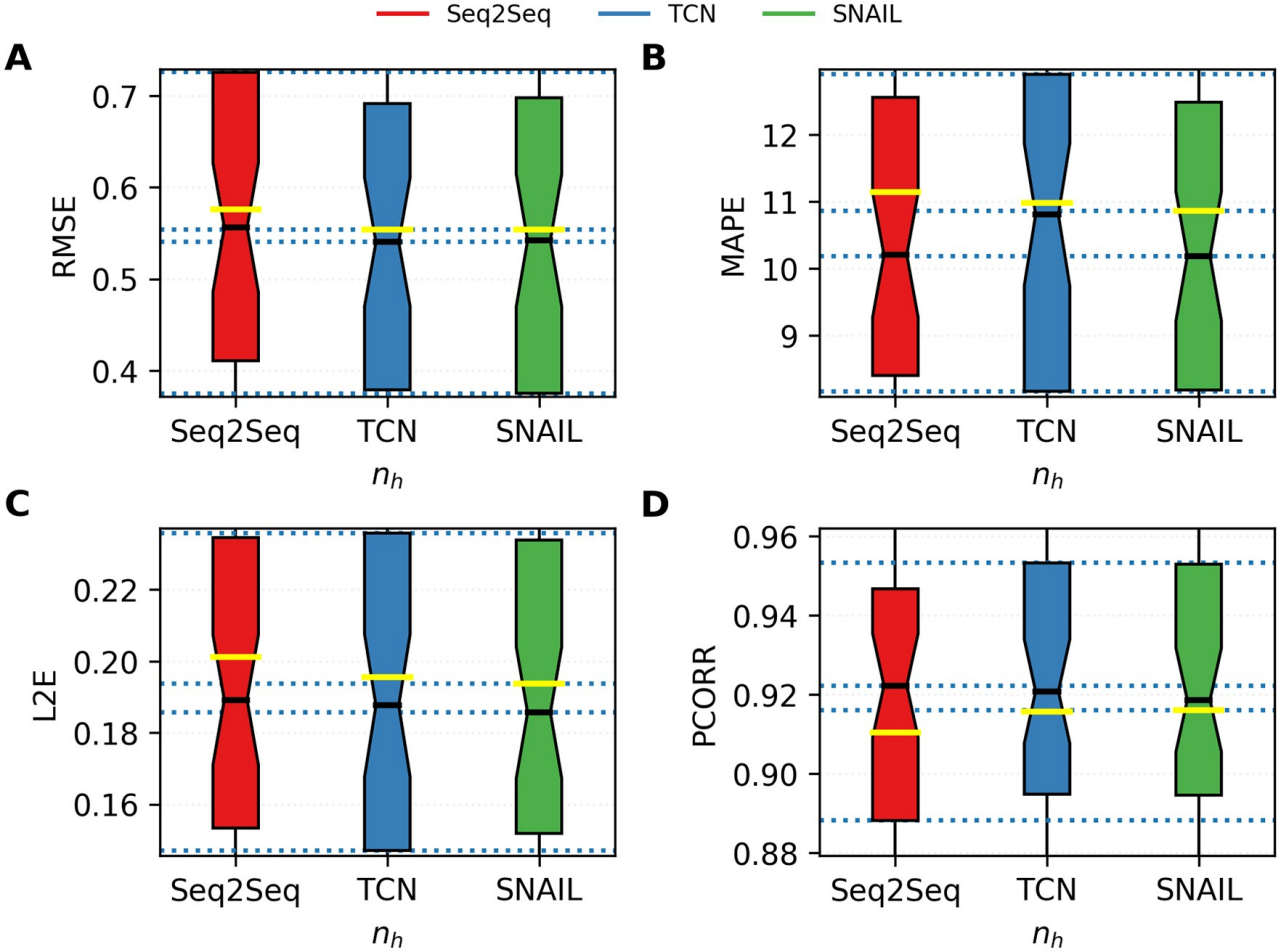
The boxplots of all four performance metrics measured by using Seq2Seq, TCN, and SNAIL. A. RMSE, B. MAPE, C. L2E, and D. PCORR. The IQRs of the boxplots are excerpted from Fig 12.

**Fig 14:**
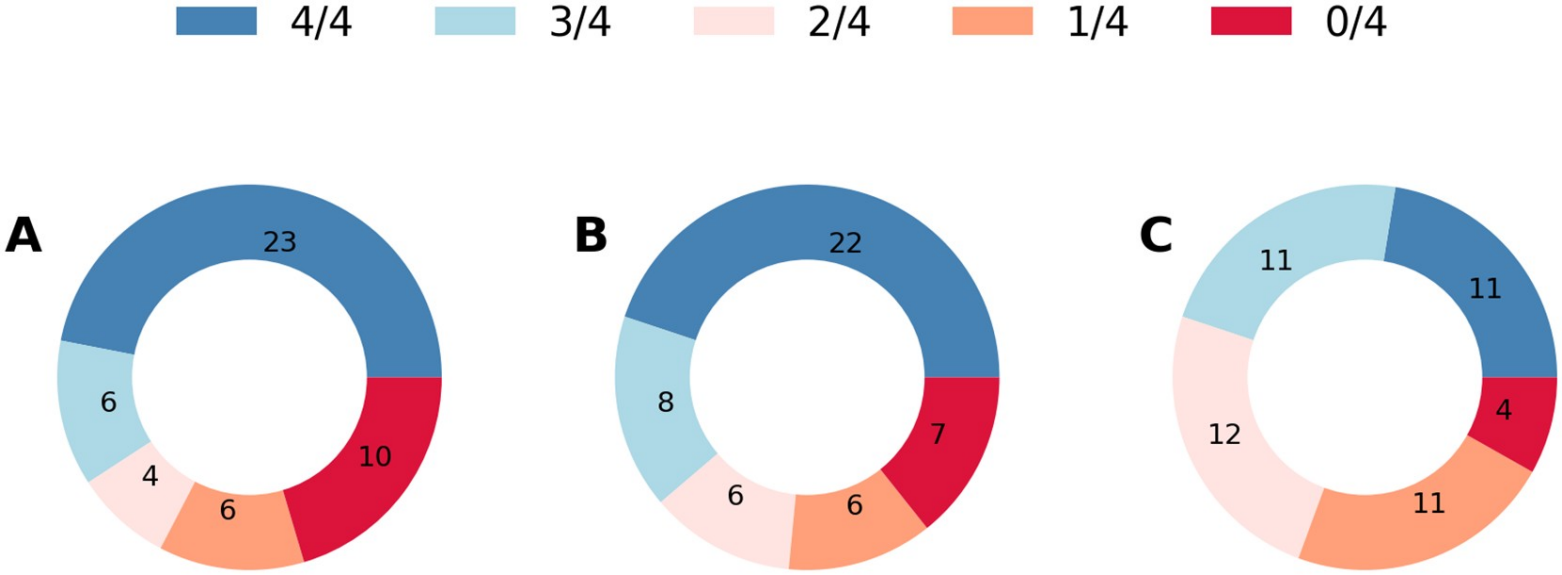
The number of the states where the former network outperforms the latter network in 0, 1, 2, 3, or all 4 metrics (RMSE, MAPE, L2E, and PCORR). A. TCN against Seq2Seq, B. SNAIL against Seq2Seq, and C. SNAIL against TCN. The numbers shown in this figure are collected from all 49 states.

Figs 12–14 provide more information on the bar plots for the results obtained with the three neural network models. Fig 12 depicts the boxplots of all four performance metrics measured by using Seq2Seq, TCN, and SNAIL. These boxplots essentially compute statistical quantities from the results shown in Fig 11: means, medians, and interquartiles ranges. Fig 14 reports the proportions of the states where TCN and SNAIL outperform Seq2Seq, respectively, and SNAIL outperforms TCN in 0, 1, 2, 3, or all 4 metrics (RMSE, MAPE, L2E, and PCORR). Both TCN and SNAIL outperform in 29 and 30 states for at least three (out of four) performance metrics than Seq2Seq. SNAIL performs slightly better than TCN; in 22 states, SNAIL performs better for at least three performance metrics, ties in 12 states, and in 15 states, TCN performs better for at least three performance metrics. These observations agree with the statistics reported in Table 3 and Fig 12.

Further, we apply Friedman’s test on the three neural network models considered above. For each error metric (i.e., RMSE, MAPE, L2E, and PCORR), we rank each models in 49 states and compute the test statistic and the *p*-value. Overall, SNAIL and TCN achieve higher ranks compared to Seq2Seq, and the test statistics for each error metric are as follows. For RMSE and L2E, the test statistic and the *p*-value are 4.204 and 0.122, respectively. For MAPE, the corresponding values are 3.959 and 0.138, respectively. As the models are trained with an objective function (and the validation as well) that measures the discrepancy between the ground truth and the predictions in mean-square error, the models are consistently ranked in RMSE and L2E. Since MAPE is a closely related error metric with MSE, the ranks of the models are also shown to be consistent over 49 states. As opposed to these three measures, for PCORR, the test statistic is 9.837 and the *p*-value is 0.007. This result suggests that the models are not consistently ranked on the error metric that the models are not directly minimized for.

**Fig 15:**
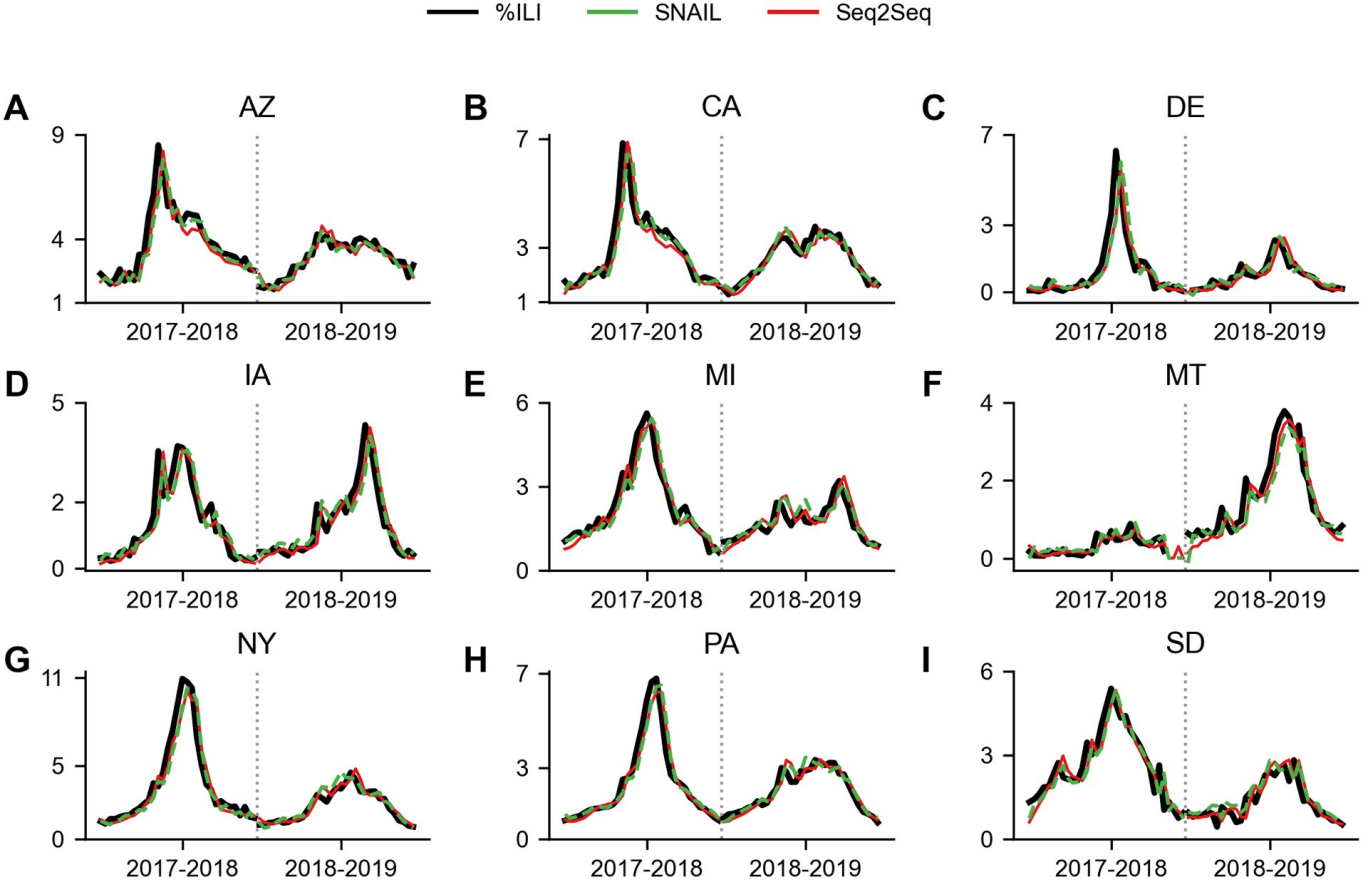
Example plots of %ILI in 9 states {AZ, CA, DE, IA, MA, MT, NY, PA, SD} and predictions made by SNAIL and Seq2Seq for two seasons 2017–2018 (validation) and 2018–2019 (test). A. Arizona, B. California, C. Delaware, D. Iowa, E. Massachusetts, F. Montana, G. New York, H. Pennsylvania, and I. South Dakota. The original %ILI is depicted in solid black lines and the prediction results of SNAIL and Seq2Seq are depicted in dashed green lines and solid red lines.

Finally, Fig 15 illustrates the original %ILI and the predictions made by Seq2Seq and SNAIL for two seasons, 2017–2018 (validation) and 2018–2019 (test), and for the nine states selected for visualization, {AZ, CA, DE, IA, MA, MT, NY, PA, SD}, which are the same states shown in Fig 15. To increase the legibility of the plots, %ILI predictions made by TCN are not reported. We note that the %ILI curves of TCN are very similar to the ones of SNAIL. A more comprehensive version of Fig 15 representing data from all 49 states, can be found in Ref. [65].

### Multi-weeks-ahead predictions

Lastly, we perform experiments with varying prediction horizons *w*_pred_. We again consider Seq2Seq, TCN, and SNAIL models. For training Seq2Seq model, as in the previous Seq2Seq setting, the *i*-th input sequence is denoted by $x_{1}^{\left(i\right)},\ldots ,x_{w_{\text{hist}}}^{\left(i\right)}$. The encoder RNN network operates on this input sequence to produce a context vector, then the decoder receives the last element in the input sequence (i.e., $x_{w_{\text{hist}}}^{\left(i\right)}$) and the context vector to produce output $\left\{o_{1}^{\left(i\right)},\ldots ,o_{w_{\text{pred}}}^{\left(i\right)}\right\}$, which attempts to match $\left\{y_{1}^{\left(i\right)},\ldots ,y_{w_{\text{hist}}}^{\left(i\right)}\right\}=\left\{x_{w_{\text{hist}}+1}^{\left(i\right)},\ldots ,x_{w_{\text{hist}}+w_{\text{pred}}}^{\left(i\right)}\right\}$. For TCN and SNAIL, *i*-th input data sequence is ${\left\{x_{\tau }^{\left(i\right)}\right\}}_{\tau =1}^{w_{\text{hist}}}$ and the *i*-th target data sequence is ${\left\{\left(y_{\tau }^{\left(i\right)},\ldots ,y_{\tau +w_{\text{pred}}-1}^{\left(i\right)}\right)\right\}}_{\tau =1}^{w_{\text{hist}}}$, where *y*_*τ*_ = *x*_*τ*+1_.

We conduct experiments for varying prediction horizons {1, 2, 3, 4} because the weekly %ILI report often takes one to four weeks to be processed and present the results in Table 4. In addition, these forecasts are used in predicting the demand for resources with long lead-times. For Seq2Seq, TCN, and SNAIL models, we pick a single configuration of hyperparameters for each model:

$$\begin{array}{cccccc} & \text{for}\;\text{Seq2Seq},\; & & \left(n_{h},w_{\text{hist}}\right) & & =(3,128),\\ & \text{for}\;\text{TCN},\; & & \left(n_{\text{R}},k,n_{k},w_{\text{hist}}\right) & & =(8,4,4,128),\\ & \text{for}\;\text{SNAIL},\; & & \left(n_{\text{TC}},k,n_{k},n_{\text{key}},n_{\text{value}},w_{\text{hist}}\right) & & =(7,8,4,32,16,128), \end{array}$$

and measure all four performance metrics with three network models.

**Table 4:** The performance metrics computed using Seq2Seq, TCN, and SNAIL for varying prediction horizons {1, 2, 3, 4}. The same sets of network hyperparameters for each model are used for varying horizons. The downward/upward arrows in parentheses indicate smaller/larger values are preferred.

	RMSE (↓)				MAPE (↓)			
horizon	1	2	3	4	1	2	3	4
Seq2Seq	0.5756	0.8453	1.037	1.052	11.28	15.55	21.73	19.55
TCN	**0.5543**	0.8117	**0.9515**	1.155	**11.09**	16.17	**18.41**	21.82
SNAIL	0.5617	**0.7915**	0.9696	**1.048**	11.16	**14.52**	20.27	**18.75**
	L2E (↓)				PCORR (↑)			
Seq2Seq	0.2012	0.2850	0.3575	0.3511	0.9096	0.8260	0.7449	**0.7463**
TCN	**0.1955**	0.2790	**0.3237**	0.3851	**0.9150**	0.8352	0.7636	0.6927
SNAIL	0.1956	**0.2667**	0.3319	**0.3479**	0.9132	**0.8435**	**0.7651**	0.7204

**Fig 16:**
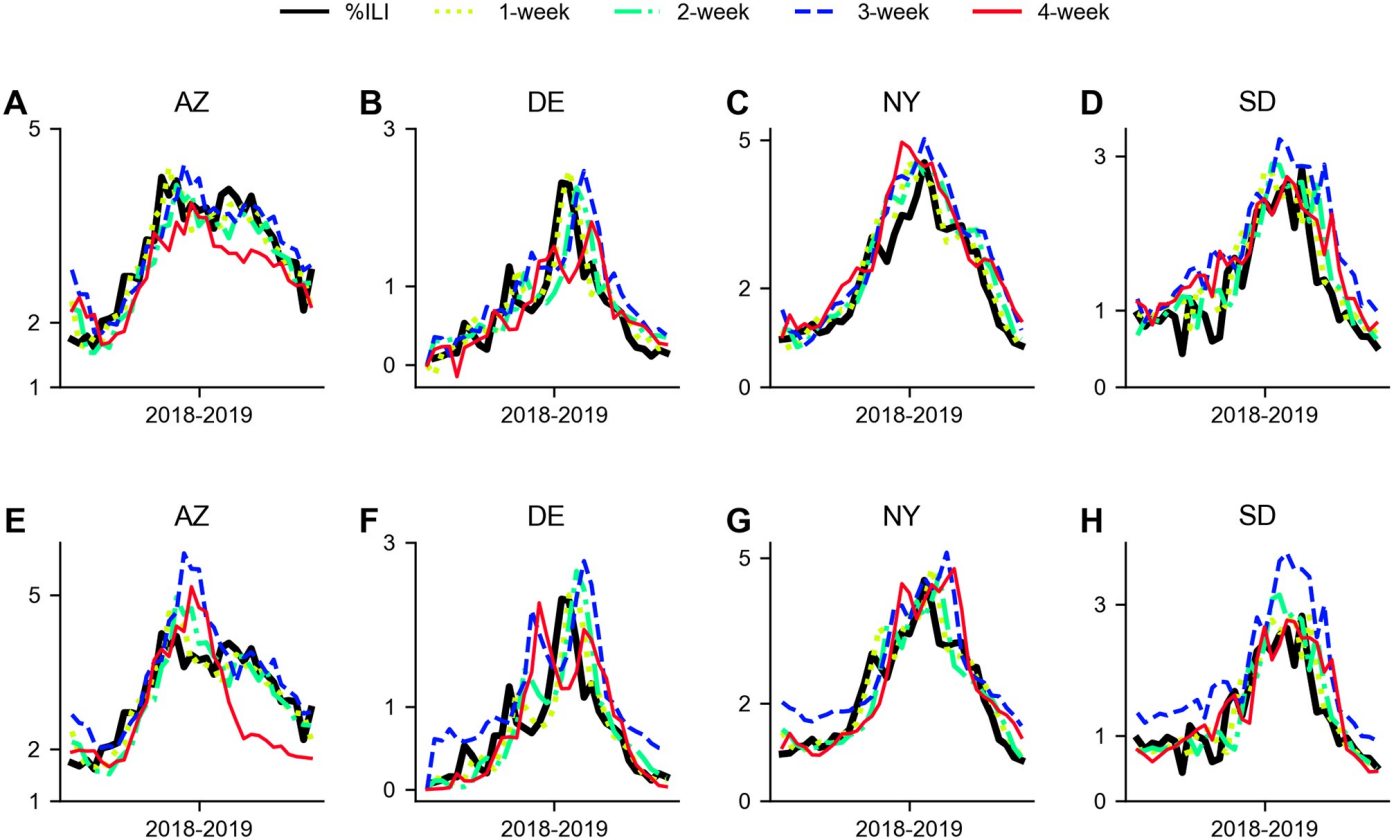
Example plots of %ILI in 4 states {AZ, DE, NY, SD} and predictions made by SNAIL and Seq2Seq for 2018–2019 (test). A. Arizona—SNAIL, B. Delaware—SNAIL, C. New York—SNAIL, D. South Dakota—SNAIL, E. Arizona—Seq2Seq, F. Delaware—Seq2Seq, G. New York—Seq2Seq, H. South Dakota—Seq2Seq. The original %ILI is depicted in solid black lines and the {1, 2, 3, 4}-weeks ahead prediction results are depicted in dotted yellow-green lines, dash-dot light green lines, dashed blue lines, and solid red lines, respectively.

In most cases, both TCN and SNAIL outperform Seq2Seq in all error metrics for *w*_pred_ = {1, 2, 3}. For *w*_pred_ = 4, SNAIL outperforms Seq2Seq in three error metrics and TCN for all error metrics and Seq2Seq outperforms TCN in all four error metrics. The prediction accuracy measured via TCN and SNAIL in all four metrics are degraded as *w*_pred_ increases from 3 to 4. Interestingly, as opposed to such degradation, there is virtually no degradation in the prediction accuracy computed using Seq2Seq as *w*_pred_ increases from 3 to 4. Fig 16 illustrates the original %ILI and the multi-weeks ahead predictions made by SNAIL and Seq2Seq for 2018—2019 season (test). Among the nine states, we select four states for visualization {AZ, DE, NY, SD}. As shown in Table 4, the prediction accuracy becomes degraded as the longer prediction horizon is taken. Nonetheless, the prediction results yield moderately accurate predictions capturing different %ILI dynamics in each state (e.g., SNAIL predicts %ILI ranges from around 1.5 to 5 in AZ, NY and from 1.5 to 3 in AZ, SD) and also capturing the useful properties such as peaks and slopes in most cases.

### Discussion

The series of experiments above show the effect of the simple, uni-modal, if noisy, influenza curves. Table 5 summarizes all neural network models considered for this study. The first class of models are RNNs with LSTM or GRU cells, where the output of each RNN step is a one-week ahead prediction. Fig 7 shows that adding multiple layers to the RNNs (irrespective of whether LSTM or GRU cells are used) only marginally improve predictive skill and training the model on a longer sequence of data (Fig 6) does not results in any improvement. That is, the RNN models achieve their predictive skill with modest data and modest architectural complexity, and training a higher capacity model results in a only a marginally better one-week-ahead forecast.

**Table 5:** Model summary table.

Recurrent neural networks	
RNN-LSTM	Stacked recurrent neural network with LSTM cells—RNN-LSTM operates on an input sequence and make the *N*-step ahead prediction at each RNN step
RNN-GRU	Stacked recurrent neural network with GRU units—RNN-GRU operates on an input sequence and make the *N*-step ahead prediction at each RNN step
Sequence-to-sequence-type neural networks	
Seq2Seq (GRU)	Encoder-decoder type architecture: both the encoder and the decoder are modeled as RNN-GRU—the encoder produces a context vector, which is then fed into the decoder RNN; the decoder reconstructs the input sequence shifted by the *N* steps.
Seq2Seq (NODE)	Encoder-decoder type architecture: the encoder is modeled as RNN-GRU and the decoder is modeled as neural ordinary differential equations—the encoder produces a context vector, which is then used as an initial condition for the NODE decoder
Temporal-convolution-layers-based neural networks	
TCN	Temporal convolutional neural networks with residual blocks: each residual block consists of a sequence of temporal convolutional layers with an increasing dilation rate; the final output is the *N*-step ahead prediction.
SNAIL	Temporal convolutional neural networks with a temporal-convolution (TC) block and attention layers: 1) the TC block consists of a series of dense blocks, with each using two parallel dilated TCs and 2) attention layers point out at which points in the input sequence should be more emphasized. The final output is the *N*-step ahead prediction.

The next class of models considered in this study is sequence-to-sequence type (or encoder-decoder type) neural networks, where the encoder takes the input sequence (history), then generates the context vector, and the decoder utilizes the context vector to make predictions. Fig 8 and Table 1 show the impact of different architectural choices (i.e., either with RNN-GRU decoder or NODE decoder) and training data size for the Seq2Seq model. We see that the improvement in predictive skill is modest: the Seq2Seq model performs slightly better than the RNN models, implying that the complexity or model-form of Seq2Seq is of the wrong type and consequently does not contribute to predictive skill. We believe that introducing more advanced deep learning techniques such as attention mechanism to Seq2Seq architecture might be a key to make improvements in prediction accuracy as shown in [16, 17]. Furthermore, we have demonstrated that having NODE or ANODE decoder in Seq2Seq architecture does not help improving the prediction accuracy. We believe this is partly because enforcing short-term dependency in NODE or ANODE is difficult as opposed to RNN-GRU.

We conclude with temporal-convolution-layers-based neural networks, where the temporal causal convolution provides a better means to incorporate distant history by using exponential increasing dilation. As opposed to RNN, which takes multiple RNN steps to convey the information contained in the distant input, TCN provides a provide more direct computational path to the distant input. The TC-layer-based models show the better outcomes in Figs 9 and 10—combinations of higher architectural complexity (i.e., *n*_R_ > 6 and *n*_TC_ > 7) and longer training sequence *w*_hist_ > 32 result in a better model. Fig 11 and Table 3 show that, on the whole, TCN and SNAIL outperform Seq2Seq. Table 4 shows a more complex behavior where both TCN and SNAIL perform better than Seq2Seq although Seq2Seq demonstrated less degradation of accuracy as the prediction horizon increases from 3 to 4 weeks.

Overall, we conclude that the TC-layer-based models achieve better performance due to their larger receptive field, which allows the models to take and process longer training sequences easily. We also note that, as shown in Ref. [17], the RNN-LSTM model, which had the least impressive performance in this study, is substantially better than ARIMA (3, 0, 3)—it has about half the RMSE, and the Pearson correlation with the data that is about 20% better. Thus, deep learning models, irrespective of RNNs or CNNs, could be profitably employed in epidemiological modeling.

## Related work

Various deep learning techniques for sequence modeling have been employed for ILI predictions. As we described in Introduction, nearly all deep-learning-based approaches heavily rely on RNNs with LSTM. We categorize these approaches into two classes: one class of approaches that utilizes only CDC data and another class of approaches that utilizes external factors affecting flu activity or external information that can be used as an indicator of flu activity.

### CDC data only

In Ref. [13], weekly influenza activity levels at week *τ* were used as an input to convolutional layers to extract features that capture spatial correlations across regions where the statistics are collected (e.g., the U.S. states) and then the extracted features were used as an input to an RNN with GRU to capture temporal correlations of the weekly influenza activity levels. Ref. [12] studied four variant constructions of LSTM networks with %ILI data: one instance is a single LSTM network, where a single application of the trained network gives multi-step-ahead predictions, and another instance is a set of multiple LSTM networks, where each LSTM is trained to predict %ILI at specific time index of multi-step-ahead predictions.

### External factors

Another line of deep-learning-based approaches is time-sequence models assisted by exogenous factors. Ref. [11] utilized features extracted from a Twitter dataset as input features to LSTM networks along with ILI information collected from military populations. Ref. [14] used climate information and geo-spatial factors as input features to LSTM networks, while Ref. [15] proposed a framework for high-resolution (e.g., county-level) ILI predictions, which generates a high-resolution synthetic dataset via an epidemic simulator and then use the dataset for training a neural network. The proposed neural network consists of two LSTM networks; one processes within-season sequences and the other processes between-season sequences. In Ref. [16], a Seq2Seq model with LSTM is trained with %ILI and Google trends data.

## Conclusion

In this study, we have investigated the feasibility of modeling epidemiological data with variants of temporal convolutional network models, and demonstrated their performance gain over recurrent neural network models. We investigate whether deep-learning models provide any advantage in forecasting skill over linear methods and perform extensive experiments for comparing all considered neural network models including RNN-LSTM, RNN-GRU, Sequence-to-Sequence with RNN-GRU decoder and neural ordinary differential equation decoder, temporal convolution networks, and simple neural attentive meta-learner. We have observed that RNN-LSTM, which has been shown to be far better than conventional models including AR and ARIMA models in our own experiments and experimental results in Refs. [15, 17] or classical machine learning methods such as SVM in Ref. [11], performs the worst among all considered neural network models and we find that neural networks based on temporal convolutional layers (TCN and SNAIL) tend to outperform RNN-LSTM/RNN-GRU and Seq2Seq models.

However, considering the significant increase in neural network complexity, the performance improvements made by TCN and SNAIL over RNN-GRU can be seen as modest, which suggests that modeling the simple, uni-modal, if noisy, form of influenza curves may not require a tremendous degree of complexity in the neural network architectures. This bottleneck may be due to an intrinsic limitation of purely data-driven approach and could be overcome by using external data or indicators of influenza-like illness or building a model that combines data-driven approach with mechanistic or compartmental models. Nonetheless, we observe from our extensive experiments that complex deep learning models can be fitted to relatively modest epidemiological data without suffering from over-fitting, thanks to our mini-batching stochastic gradient optimizer with the early-stopping strategy, and can provide better forecasts than conventional data-driven ARIMA models and simple RNN-GRU models.

## Supporting information

S1 Appendix Detailed technical descriptions of neural network architectures. (PDF)
