# Supplementary material for: The predictive skill of convolutional neural networks models for disease forecasting
Source: PLoS One. 2021 Jul 9;16(7):e0254319. doi: 10.1371/journal.pone.0254319 (PMC8270135; doi:10.1371/journal.pone.0254319)
Supplement: S1 Appendix — (PDF) [file pone.0254319.s001.pdf]

S1 Appendix: detailed technical descriptions of neural network architectures

Kookjin Lee, Jaideep Ray, and Cosmin Safta

Long short-term memory

The detailed operations of an LSTM model are described here.

Forget gate: The forget gate controls the extent to which internal cell state remains. The forget gate takes the input data $\boldsymbol{x}_\tau$ and the hidden state $\boldsymbol{h}_{\tau-1}$ from the previous cell as the input and then produces a value between [0, 1] with the element-wise sigmoid nonlinear function $\sigma(\cdot)$ such that

$$\boldsymbol{f}_\tau = \sigma(\boldsymbol{b}^f + U^f \boldsymbol{x}_\tau + W^f \boldsymbol{h}_{\tau-1}),$$

where $\boldsymbol{b}^f$ is biases and U^f, W^f are network weights of the forget gate.

Input gate: The input gate controls the extent to which the new input data $\boldsymbol{x}_\tau$ is forwarded to produce a new LSTM cell state. The input gate also takes the input data $\boldsymbol{x}_\tau$ and the hidden state $\boldsymbol{h}_{\tau-1}$ from the previous cell as the input and then produces a value between [0, 1] with the element-wise sigmoid nonlinear function $\sigma(\cdot)$ such that

$$\boldsymbol{g}_\tau = \sigma(\boldsymbol{b}^g + U^g \boldsymbol{x}_\tau + W^g \boldsymbol{h}_{\tau-1}),$$

where $\boldsymbol{b}^g$ is biases and U^g, W^g are network weights of the forget gate.

Together with the forget gate and the input gate, the new cell state $\boldsymbol{s}_\tau$

$$\boldsymbol{s}_\tau = \boldsymbol{f}_\tau \odot \boldsymbol{s}_{\tau-1} + \boldsymbol{g} \odot \sigma(\boldsymbol{b} + U\boldsymbol{x}_\tau + W\boldsymbol{h}_{\tau-1}), \qquad (1)$$

where $\boldsymbol{b}, U$, and W are biases and weights to the LSTM cell and $\odot$ denotes element-wise multiplications.

Output gate: The output gate controls the extent to which the cell output flows out to the output using the similar gating mechanism using the sigmoid activation function such that

$$\boldsymbol{q}_\tau = \sigma(\boldsymbol{b}^q + U^q \boldsymbol{x}_\tau + W^q \boldsymbol{h}_{\tau-1}),$$

where $\boldsymbol{b}, U^q$, and W^q are biases and weights of the output gate, and the output of the LSTM cell computed as

$$\boldsymbol{h}_\tau = \tanh(\boldsymbol{s}_\tau) \odot \boldsymbol{q}_\tau.$$

Fig 1 illustrates the diagram of a LSTM cell. The MLP block stands for the second operand of the second term on the right-hand side (1).

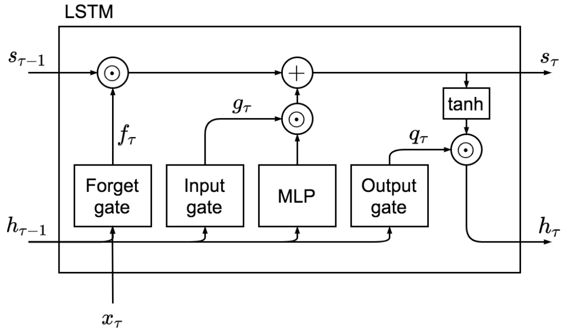

Figure 1: LSTM cell diagram.

Gated recurrent unit

The detailed operations of GRU are described below. GRU does not have an additional cell state as in LSTM and consists of the two gating units, the update gate and the reset gate. The output of the GRU is computed as follows:

$$\boldsymbol{h}_\tau = (1 - \boldsymbol{u}_\tau) \odot \boldsymbol{h}_{\tau-1} + \boldsymbol{u}_\tau \odot \sigma(\boldsymbol{b} + U\boldsymbol{x}_\tau + W(\boldsymbol{r}_\tau \odot \boldsymbol{h}_{\tau-1})), \tag{2}$$

where the values of the update gate and the reset gate are determined as

$$\boldsymbol{u}_\tau = \sigma(\boldsymbol{b}^u + U^u\boldsymbol{x}_\tau + W^u\boldsymbol{h}_\tau)$$

and

$$\boldsymbol{r}_\tau = \sigma(\boldsymbol{b}^r + U^r\boldsymbol{x}_\tau + W^r\boldsymbol{h}_\tau).$$

Fig 2 illustrates the diagram of GRU. Analogous to the LSTM diagram, the MLP block stands for the second operand of the second term on the right-hand side (2). Morever, 1- block computes $1 - \boldsymbol{u}_{\tau-1}$.

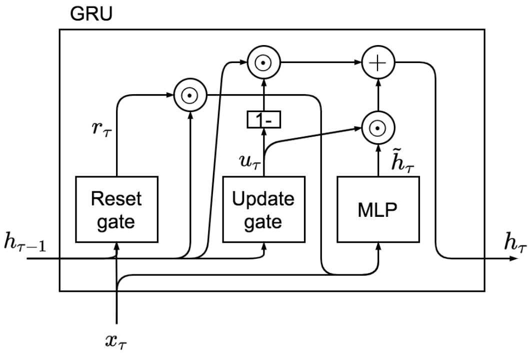

Figure 2: GRU cell diagram.

Comparison between LSTM and GRU As the formulation shows GRU has the simpler architecture than LSTM; it does not have the cell state and only two gates. The update gate combines the role of the forget gate and the input gate, which results in simpler recurring network architectures with less network parameters. Thus, LSTM performs better in many applications, not just large-scale applications [1], but also in rather simple applications [2]. This does not mean, however, that LSTM always performs better than GRU. In certain tasks, including natural language processing, speech signal modeling, GRU performs similar to LSTM [3], or even performs better in small datasets [4].

Neural Ordinary Differential Equations

Neural ordinary differential equations (ODEs) [5–7] introduce a new family of deep neural network models, which learn time-continuous dynamics of hidden states by parameterizing the dynamics of hidden states using an ODE; the derivatives of the states are specified by a neural network,

$$\frac{d\boldsymbol{h}(t)}{dt} = f(\boldsymbol{h}(t), t; \Theta), \qquad \boldsymbol{h}(0) = \boldsymbol{h}^0 \tag{3}$$

where the dynamics f is parameterized by Θ. The dynamics f is often modeled as an MLP with hyperbolic tangent nonlinearity and Θ corresponds to a collection of network weights and biases. In the forward pass, a black-box differential solver can be used to solve an initial value problem defined by Eq. 3. In the backward pass, the network parameters Θ can be learned by a back-propagation method; in particular, as proposed in Ref. [6], gradients can be computed by solving another system of ODEs, which are derived using the adjoint-sensitivity method [8]. We refer Ref. [6] for the complete description of the back-propagation process.

As NODEs model dynamical systems described by a system of ODEs, they have been applied to many time-sequence modeling studies to make predictions of future states and have demonstrated their effectiveness: to name a few, example applications include turbulence forecasting [9], climate forecasting [10], predicting health conditions of patients obtained from intensive care unit [11], and human activity [11,12].

NODEs, however, learn a single dynamic for all data in a given distribution and they have a limited capability to learn diverse dynamics [13]. To mitigate this issue, the authors in [14] proposed augmented NODEs (ANODEs), which are more expressive, more stable, and generalize better than the original neural ODE. ANODEs augment extra dimensions to the states space of ODEs

$$\frac{\mathrm{d}}{\mathrm{d}t}\begin{bmatrix}\boldsymbol{h}(t)\\ \boldsymbol{a}(t)\end{bmatrix} = f\left(\begin{bmatrix}\boldsymbol{h}(t)\\ \boldsymbol{a}(t)\end{bmatrix}, t; \Theta\right), \qquad \begin{bmatrix}\boldsymbol{h}(0)\\ \boldsymbol{a}(0)\end{bmatrix} = \begin{bmatrix}\boldsymbol{h}^0\\ \boldsymbol{0}\end{bmatrix},$$

so that the learned model can be more expressive. The authors also hypothesize that the learned f from the augmented space is smoother, which leads to efficient computations (i.e., requiring fewer steps in ODE solvers). Analogous to NODEs, in the forward pass, a black-box differential solver can be used and, in the backward pass, the adjoint-sensitivity method can be used for the back-propagation.

Motivated by the successful applications of NODEs and ANODEs to various prediction/forecasting tasks, we test NODEs and ANODEs for state-level %ILI predictions. Following Refs. [11, 12], we consider Seq2Seq architecture, where NODEs or ANODEs are used to model a decoder, which takes a context vector as an initial condition $\boldsymbol{h}^0$, produces hidden states in future time steps, and the resulting hidden states are fed to an MLP to produce a final output. To compute the hidden states at specified time indices $\{\bar{\boldsymbol{h}}(t_1), \ldots, \bar{\boldsymbol{h}}(t_N)\}$, we use a black-box differential equation solver, i.e.,

$$\{\bar{\boldsymbol{h}}(t_1), \ldots, \bar{\boldsymbol{h}}(t_N)\} = \mathrm{ODESolve}(\bar{\boldsymbol{h}}(0), f_\Theta, t_1, \ldots, t_N),$$

where $\bar{\boldsymbol{h}}(t) = [\boldsymbol{h}(t), \boldsymbol{a}(t)]^\mathsf{T}$ for ANODEs and $\bar{\boldsymbol{h}}(t) = \boldsymbol{h}(t)$ for NODEs. In the experiments, for both NODEs and ANODEs, we choose the Dormand–Prince time integrator [15].

Temporal Convolutional Network

The detailed operations of TCN are described below. Let us denote a 1-dimensional input sequence by $\boldsymbol{x} \in \mathbb{R}^n$ and the kernel filter by $\boldsymbol{f} \in \mathbb{R}^k$.

Causal convolution The causal convolution is the operator that can only look at the current time $t = \tau$ input data and its previous data $(t < \tau)$, which can be formally written as

$$(\boldsymbol{x} * \boldsymbol{f})(\tau) = \sum_{i=0}^{k-1} \boldsymbol{f}_i \cdot \boldsymbol{x}_{\tau-i},$$

where $*$ defines the convolution between two sequences. This expression shows that to obtain an output at $t = \tau$, the convolution operator only looks at the data at $t \leq \tau$.

Dilated causal convolution The dilated causal convolution increases the size of receptive fields by adding dilation factor, which is denoted by d:

$$(\boldsymbol{x} *_d \boldsymbol{f})(\tau) = \sum_{i=0}^{k-1} \boldsymbol{f}_i \cdot \boldsymbol{x}_{\tau-d\cdot i}.$$

The convolution operator $*_d$ takes every d element of the input $\boldsymbol{x}$. Thus, a series of dilated causal convolutions with increasing dilation factor $d = 2^\ell$ can take a receptive field of size dk.

ResBlock TCN employs the residual block consisting of two layers of dilated causal convolutions, weight normalization, ReLU, and Dropout, and an identity mapping from the input. Formally, ResBlock can be written as

$$\mathsf{z}_{i+1} = \mathsf{z}_i + \mathcal{F}(\mathsf{z}_i),$$

where z_i and z_{i+1} are the input and the output of ResBlock, and $\mathcal{F}(\cdot)$ is a function consisting of the two layers of dilated causal convolutions.

Fig 3 illustrate the flow of computations performed in one residual block. In the residual block, two dilated convolutional layers share the same hyperparameters (i.e., kernel size k, dilation factor d, and the number of channels n_k). By stacking the residual blocks with increasing dilation factors d, the size of the receptive field can grow exponentially.

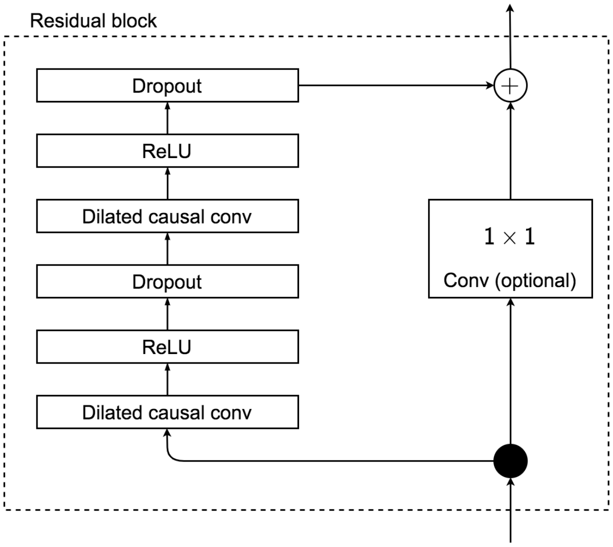

Figure 3: TCN ResBlock. The black circle indicates a point where the input takes two separate and parallel paths. The residual block consists of two layers of dilated convolutional layers, each of which followed by weight normalization, ReLU, and Dropout. The weight normalization is applied to each convolutional filters in the forward pass. Then the resulting quantity of the dilated convolutions and the input are summed element-wise. If the input and the output sizes do not match, an optional 1×1 convolution can be used. Otherwise, it is an identity mapping.

Simple neural attentive meta-learner

The detailed operations of SNAIL are described below.

SNAIL – TCBlock The main component of the TCBlock is also one-dimensional dilated causal convolutions. TCBlock consists of a series of multiple DenseBlocks with increasing dilation factors. The number of DenseBlocks in one TCBlock is determined by the input sequence length n (i.e., $\lceil n \rceil$). That is, TCBlock ensures that the size of receptive field covers the entire input sequence. The ℓth DenseBlock consists of dilated causal convolutions with dilation factors $2^{\ell-1}$. In the original paper, the dilation factor for the ℓth DenseBlock is 2^{ℓ}. DenseBlock consists of two parallel dilated causal convolutions with the same hyperparameter settings (i.e., kernel size k, dilation factor d, and the number of channels n_k), but followed by different nonlinear activations tanh and sigmoid. The outputs of the convolutional layers are multiplied element-wise and then the resulting quantity is concatenated with the input. Fig. 4 illustrates the computation flow of DenseBlock.

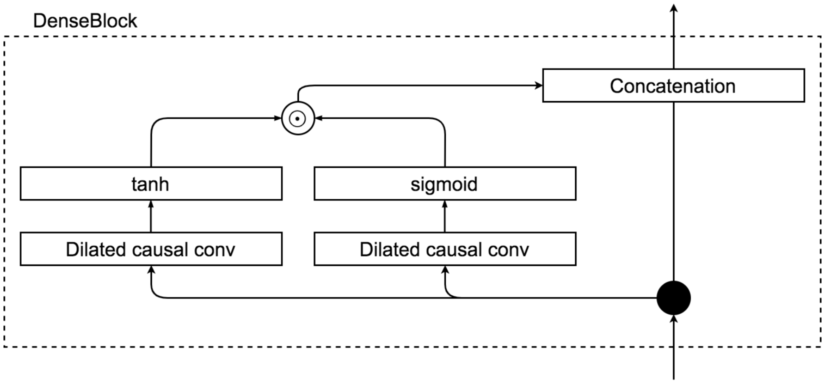

Figure 4: SNAIL DenseBlock. The input takes two separate and parallel paths at the point indicated by the black circle. Then DenseBlock applies two parallel dilated causal convolutions and the outputs of the convolutional layers are multiplied element-wise. Finally, the resulting quantity of the element-wise multiplication is concatenated with the input.

SNAIL – AttentionBlock AttentionBlock is designed to perform the key-value-pair-based *self-attention* mechanism proposed by [16]. The self-attention mechanism refer to a design which learns relations of elements in different positions of a single sequence. The self-attention mechanism employed in SNAIL is a *soft-attention* mechanism because the relations of entire elements in a sequence are considered. This is in contrast to a *hard-attention* mechanism, where the mechanism attends to a specific region (e.g., a small patch in an image).

The key-value-pair-based attention mechanism is operated on three quantities: *key*, *value*, and *query*. When *query* is given, the attention mechanism first attempts to match *key*, which is associated with *value* that is closest to what the query is looking for. In SNAIL, all three quantities are computed by applying a single-layer feed-forward network to the input of AttentionBlock, which is usually the hidden states produced by TCBlock. AttentionBlock first applies three separate affine transformations to the input to obtain V (*value*), Q (*query*), and K (*key*). Then AttentionBlock finds how relevant *query* Q and *key* K by using the scaled-dot product $\frac{\mathrm{QK}^\mathsf{T}}{\sqrt{n_{\mathrm{key}}}}$ and by applying causally masked softmax to convert the resulting quantity into the probability. Here, the causal mask prevents the τth query from accessing future key/values, i.e., $P = \mathrm{softmax}\left(M \odot \frac{\mathrm{QK}^\mathsf{T}}{\sqrt{n_{\mathrm{key}}}}\right)$, where M is the causal mask whose upper triangular part is zeroed out. Then multiplying P with V finally gives target *values* that *queries* look for. Fig. 5 illustrates the AttentionBlock computation flow.

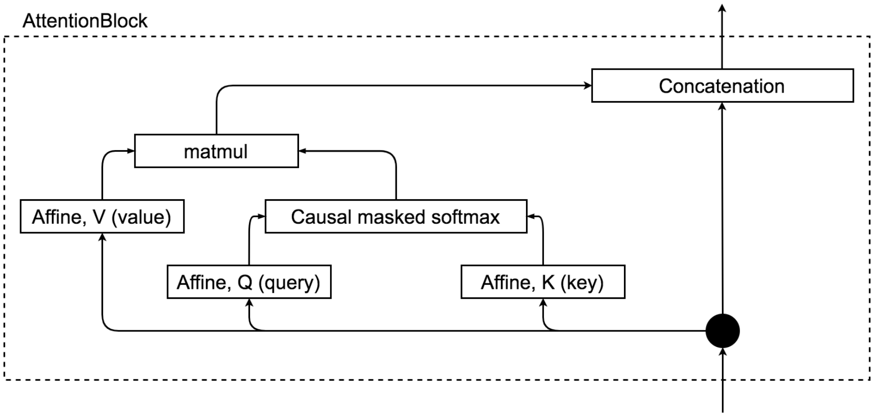

Figure 5: SNAIL AttentionBlock. The input takes two separate and parallel paths at the point indicated by the black circle. Then AttentionBlock computes *key*, *value*, and *query* from the input of AttentionBlock, and processes *query* to find *key* associated with *value*, which is the closest to what *query* is looking for. From *key* and *query*, *probability* is computed from softmax and, subsequently, is multiplied with *value* to find the most likely target *value*. Finally, the resulting quantity (i.e., target *value*) is concatenated with the input.

References

[1] Britz D, Goldie A, Luong MT, Le Q. Massive exploration of neural machine translation architectures. arXiv preprint arXiv:170303906. 2017;.

[2] Weiss G, Goldberg Y, Yahav E. On the practical computational power of finite precision RNNs for language recognition. arXiv preprint arXiv:180504908. 2018;.

[3] Ravanelli M, Brakel P, Omologo M, Bengio Y. Light gated recurrent units for speech recognition. IEEE Transactions on Emerging Topics in Computational Intelligence. 2018;2(2):92–102.

[4] Chung J, Gulcehre C, Cho K, Bengio Y. Empirical evaluation of gated recurrent neural networks on sequence modeling. arXiv preprint arXiv:14123555. 2014;.

[5] Weinan E. A proposal on machine learning via dynamical systems. Communications in Mathematics and Statistics. 2017;5(1):1–11.

[6] Chen TQ, Rubanova Y, Bettencourt J, Duvenaud DK. Neural ordinary differential equations. In: Advances in Neural Information Processing Systems; 2018. p. 6571–6583.

[7] Ruthotto L, Haber E. Deep neural networks motivated by partial differential equations. Journal of Mathematical Imaging and Vision. 2019; p. 1–13.

[8] Pontryagin LS. The mathematical theory of optimal processes. 1962;.

[9] Portwood GD, Mitra PP, Ribeiro MD, Nguyen TM, Nadiga BT, Saenz JA, et al. Turbulence forecasting via Neural ODE. arXiv preprint arXiv:191105180. 2019;.

[10] De Brouwer E, Simm J, Arany A, Moreau Y. GRU-ODE-Bayes: Continuous modeling of sporadically-observed time series. In: Advances in Neural Information Processing Systems; 2019. p. 7379–7390.

[11] Rubanova Y, Chen RT, Duvenaud DK. Latent ordinary differential equations for irregularly-sampled time series. In: Advances in Neural Information Processing Systems; 2019. p. 5320–5330.

[12] Yildiz C, Heinonen M, Lahdesmaki H. ODE2VAE: Deep generative second order ODEs with Bayesian neural networks. In: Advances in Neural Information Processing Systems; 2019. p. 13412–13421.

[13] Chalvidal M, Ricci M, VanRullen R, Serre T. Neural optimal control for representation learning. arXiv preprint arXiv:200609545. 2020;.

[14] Dupont E, Doucet A, Teh YW. Augmented neural ODEs. In: Advances in Neural Information Processing Systems; 2019. p. 3134–3144.

[15] Dormand JR, Prince PJ. A family of embedded Runge–Kutta formulae. Journal of Computational and Applied Mathematics. 1980;6(1):19–26.

[16] Vaswani A, Shazeer N, Parmar N, Uszkoreit J, Jones L, Gomez AN, et al. Attention is all you need. In: Advances in neural information processing systems; 2017. p. 5998–6008.
